# Mapping glycoprotein structure reveals *Flaviviridae* evolutionary history

**DOI:** 10.1038/s41586-024-07899-8

**Published:** 2024-09-04

**Authors:** Jonathon C. O. Mifsud, Spyros Lytras, Michael R. Oliver, Kamilla Toon, Vincenzo A. Costa, Edward C. Holmes, Joe Grove

**Affiliations:** 1 Sydney Institute for Infectious Diseases, School of Medical Sciences, The University of Sydney, Sydney, New South Wales Australia; 2 MRC–University of Glasgow Centre for Virus Research, Glasgow, UK; 3 Division of Systems Virology, Department of Microbiology and Immunology, The Institute of Medical Science, The University of Tokyo, Tokyo, Japan; 4 Laboratory of Data Discovery for Health Limited, Hong Kong SAR, China

**Keywords:** Viral membrane fusion, Protein structure predictions, Viral evolution

## Abstract

Viral glycoproteins drive membrane fusion in enveloped viruses and determine host range, tissue tropism and pathogenesis^1^. Despite their importance, there is a fragmentary understanding of glycoproteins within the *Flaviviridae*^2^, a large virus family that include pathogens such as hepatitis C, dengue and Zika viruses, and numerous other human, animal and emergent viruses. For many flaviviruses the glycoproteins have not yet been identified, for others, such as the hepaciviruses, the molecular mechanisms of membrane fusion remain uncharacterized^3^. Here we combine phylogenetic analyses with protein structure prediction to survey glycoproteins across the entire *Flaviviridae*. We find class II fusion systems, homologous to the Orthoflavivirus E glycoprotein in most species, including highly divergent jingmenviruses and large genome flaviviruses. However, the E1E2 glycoproteins of the hepaciviruses, pegiviruses and pestiviruses are structurally distinct, may represent a novel class of fusion mechanism, and are strictly associated with infection of vertebrate hosts. By mapping glycoprotein distribution onto the underlying phylogeny, we reveal a complex evolutionary history marked by the capture of bacterial genes and potentially inter-genus recombination. These insights, made possible through protein structure prediction, refine our understanding of viral fusion mechanisms and reveal the events that have shaped the diverse virology and ecology of the *Flaviviridae*.

## Abstract (web summary)

Combined phylogenetic analyses and glycoprotein structure prediction across the *Flaviviridae* reveals divergent membrane fusion systems and provides insights into the complex evolutionary history within this family of viruses.

## Main

The *Flaviviridae*^2^ is a highly diverse family of enveloped positive-sense RNA viruses that includes important pathogens of humans (for example, dengue virus, Zika virus and hepatitis C virus) and other animals (for example, classical swine fever virus and bovine viral diarrhoea virus), as well as many viruses that pose emerging threats to human health (for example, West Nile virus, Alongshan virus and Haseki tick virus^4–6^). The *Flaviviridae* is currently classified into four genera: Orthoflavivirus, Pestivirus, Pegivirus and Hepacivirus^7^. In recent years a remarkable diversity of novel flaviviruses with varied genome structures have been discovered, including the jingmenvirus group, which are unique in being both segmented and potentially multicomponent^8,9^. Another group, tentatively known as the large genome flaviviruses (LGF) are primarily associated with invertebrates^10^, but have also been linked to plants^11,12^ and vertebrates^6^. LGFs have genomes up to 39.8 kb in length, challenging previous assumptions about the maximum genome size achievable by RNA viruses that lack proofreading mechanisms^13,14^. The jingmenviruses and LGFs have yet to receive taxonomic ratification, and consensus is lacking regarding their placement within the flavivirus phylogeny.

Previous efforts to reconstruct the evolutionary history of large and diverse families of RNA viruses such as the *Flaviviridae* have relied largely on the phylogenetic analysis of highly conserved viral proteins, most notably the RNA-dependent RNA polymerase^10,15^ (RdRp). Although of considerable utility, the functions and features that define virus biology and pathogenesis are typically encoded by highly divergent sequences outside of the conserved replication machinery. In these regions, it is difficult to detect deep sequence homology and hence perform reliable multiple sequence alignment (MSA) or phylogenetic analysis. As a consequence, our understanding of long-term virus evolution is generally based on the analysis of a single protein (the RdRp), such that we lack an understanding of genome-wide relationships and hence of the genesis and evolution of viral genera and species.

Glycoproteins are likely to be important determinants of phenotypic characteristics across the *Flaviviridae*. They are essential for virus entry, influence host range and spillover potential, and are primary targets for host immune responses. However, glycoproteins have yet to be identified and/or classified for many species in the *Flaviviridae*^3^. Owing to high levels of sequence divergence, this cannot be resolved by even the most sensitive of sequence-based approaches^13,16^, and classical structural biology lacks the speed and scalability to sample enough species. This knowledge gap limits the investigation of molecular mechanisms, which in turn hinders the development of interventions such as vaccines.

Here we have augmented conventional phylogenetics with machine learning-enabled protein structure prediction to comprehensively map glycoprotein structures across the *Flaviviridae*. This provides an evolutionary and genomic-scale perspective of the entire family, revealing molecular signatures that define the diverse virology and ecology found within the *Flaviviridae*.

## *Flaviviridae* contains three major clades

**Extended Data Fig. 1:**
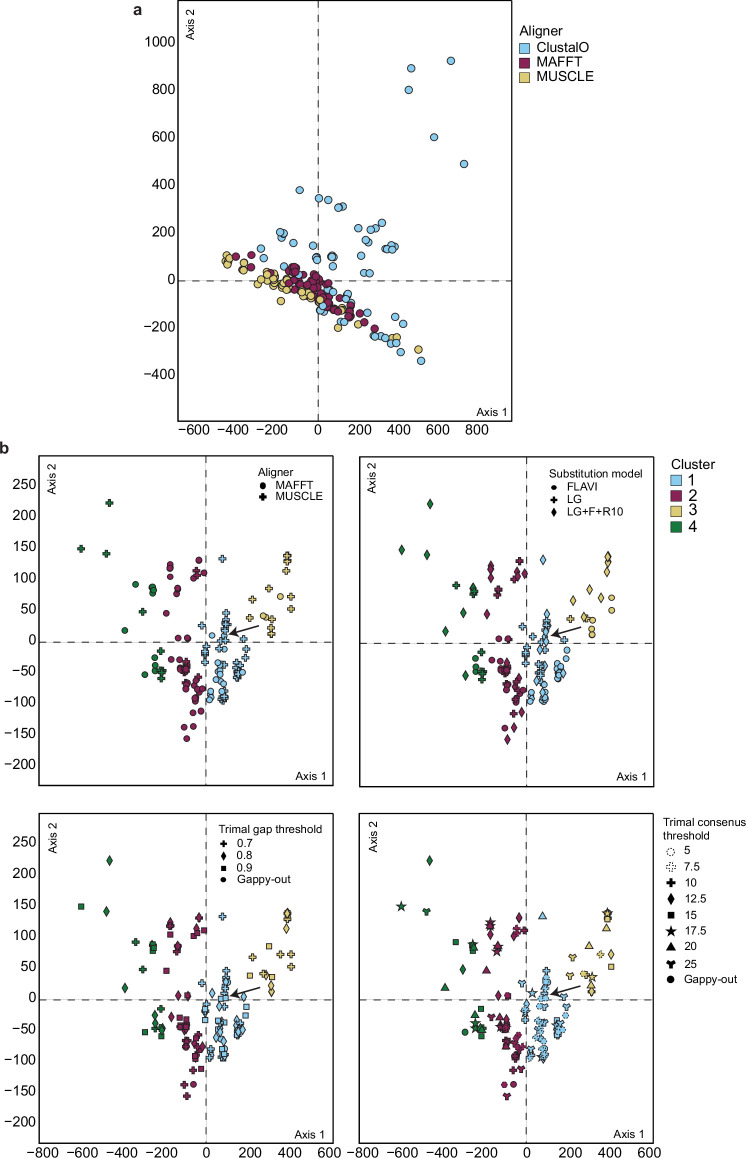
Two-dimensional MDS plot of the NS5b phylogeny variations. **a**, Coloured by alignment method **b**, Excluding trees generated from Clustal Omega alignments. Points, which represent individual phylogenies, are colour-coded based on the clusters identified using the ‘findGroves’ function. Point shapes indicate the alignment software, the trimAl gap and consensus thresholds, and the substitution model applied. An arrow signifies the master phylogeny (Tree 18) chosen for further analysis.

Understanding the evolution of molecular features across the *Flaviviridae* requires a proper gauge of the phylogenetic and genomic diversity of this family. To achieve this, we first constructed a comprehensive data set of flavivirus sequences, which after clustering and manual curation, comprised 458 flavivirus genomes with complete coding sequences, including 11 that were novel taxa identified in this study (Supplementary Table 1). We next inferred a robust family-level phylogenetic tree for these data. Using conserved NS5 gene sequences that encode the RdRp, we applied various sequence alignment methods, quality trimming protocols and amino acid substitution models, to infer a total of 225 phylogenetic trees for this family (Supplementary Table 2). Using distance-based approaches and manual inspection of the alignments and trees, we identified a phylogeny, denoted Tree 18 (Extended Data Fig. 1, Supplementary Table 2 and Supplementary Fig. 1), that appeared to best represent the consensus topology of the *Flaviviridae*. Specifically, the topological placement of the major *Flaviviridae* clades in Tree 18 was consistent with 93% of phylogenies derived from MUSCLE and MAFFT MSAs, although this percentage dropped to 70% when Clustal Omega was included (Methods).

**Fig. 1:**
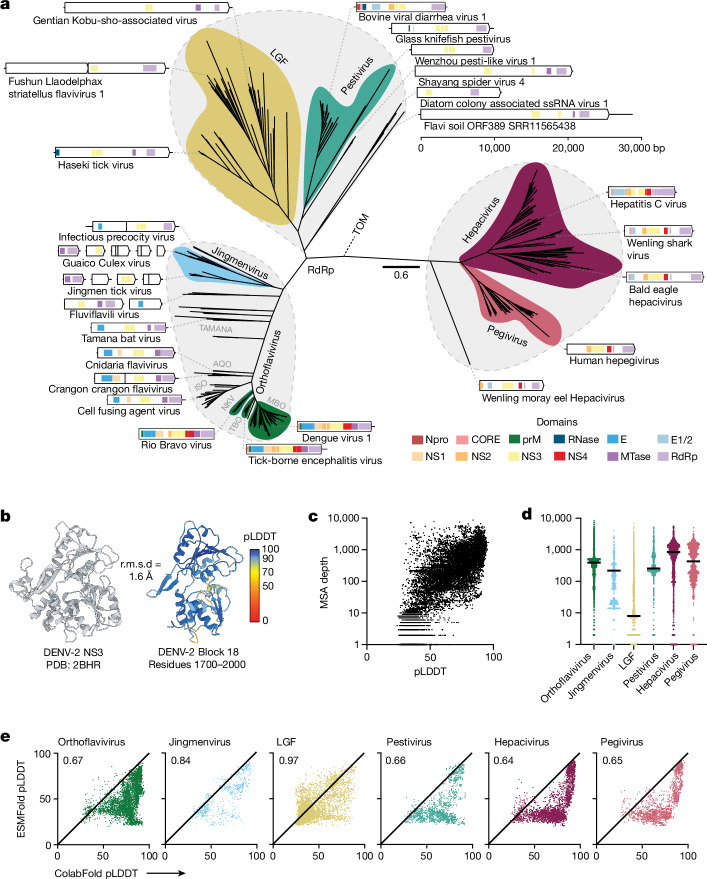
Generation of a protein foldome for the *Flaviviridae.* **a**, RdRp phylogeny reveals three major lineages within the *Flaviviridae*: (1) Orthoflavivirus/jingmenvirus (including orthoflavivirus-like—for example, Tamana bat virus); (2) LGF/Pestivirus; and (3) Hepacivirus/Pegivirus. An unrooted tree is shown, with the tombusviruses (TOM) representing the outgroup taxa and a scale bar denoting the number of amino acid substitutions per site. Genome organization is provided for exemplar species, with annotations based on InterProScan searches. **b**, Crystal structure of DENV-2 NS3 (left) shown alongside a ColabFold predicted structure for the corresponding region of the polyprotein (right). These structures superpose with a root mean square deviation (r.m.s.d.) of 1.6 Å. The predicted structure is colour-coded by per residue confidence scores (predicted local distance difference test (pLDDT)), as indicated in the bar. **c**, Scatter plot of MSA depth and prediction confidence (pLDDT). **d**, MSA depths for each sequence block in each genus or subclade, colour-coded as in **a** (orthoflavivirus-like viruses are included with the orthoflaviviruses). The mean is shown as a solid black line (*n* = 4,754 (Orthoflavivirus), 704 (jingmenvirus), 4,358 (LGF), 1,278 (Pestivirus), 2,904 (Hepacivirus) and 1,623 (Pegivirus) sequence blocks). **e**, Scatter plots representing prediction confidence (pLDDT) for ColabFold and ESMFold for each sequence block in each genus or subclade. Numerical values provide the performance ratio between the protein structure prediction methods; values below 1 indicate better performance by ColabFold.

Our best-fit RdRp phylogeny supported the division of the *Flaviviridae* into three distinct clades: (1) an Orthoflavivirus*/*jingmenvirus group (that also contains ‘orthoflavirus-like’ viruses—for example, Cnidaria flavivirus and Tamana bat virus); (2) a clade comprising the large genome flaviviruses and members of the genus Pestivirus; and (3) a Pegivirus/Hepacivirus clade (Fig. 1a). Regardless of whether the tree was unrooted or rooted on a *Tombusviridae* outgroup, the LGF/Pestivirus and Orthoflavivirus*/*jingmenvirus groups clustered together and formed a sister group to the Pegivirus/Hepacivirus clade. The Orthoflavivirus*/*jingmenvirus clade had the largest number of taxa (*n* = 182), followed by Pegivirus/Hepacivirus (*n* = 157) and LGF/Pestivirus (*n* = 119). All novel taxa (*n* = 11) identified in this study fell within the LGF/Pestivirus clade.

## Building a *Flaviviridae* protein foldome

We next aimed to explore protein functionality across the *Flaviviridae* using machine learning-enabled protein structure prediction. All flaviviruses encode polyproteins that undergo proteolytic maturation to liberate the constituent viral proteins. However, incomplete and ambiguous genome annotations combined with extensive sequence divergence make it very difficult to reliably identify the regions encoding each mature protein in all species. We therefore took a genome-agnostic approach, in which polyprotein coding sequences were split into sequentially overlapping 300-residue blocks for structural inference by two leading prediction models—ColabFold–AlphaFold2 (hereafter referred to as ColabFold) and ESMFold^17–19^. This provided a comprehensive survey of protein structure across the *Flaviviridae* (458 species, more than 16,000 sequence blocks and more than 33,000 predicted structures), referred to here as the ‘protein foldome’.

**Extended Data Fig. 2:**
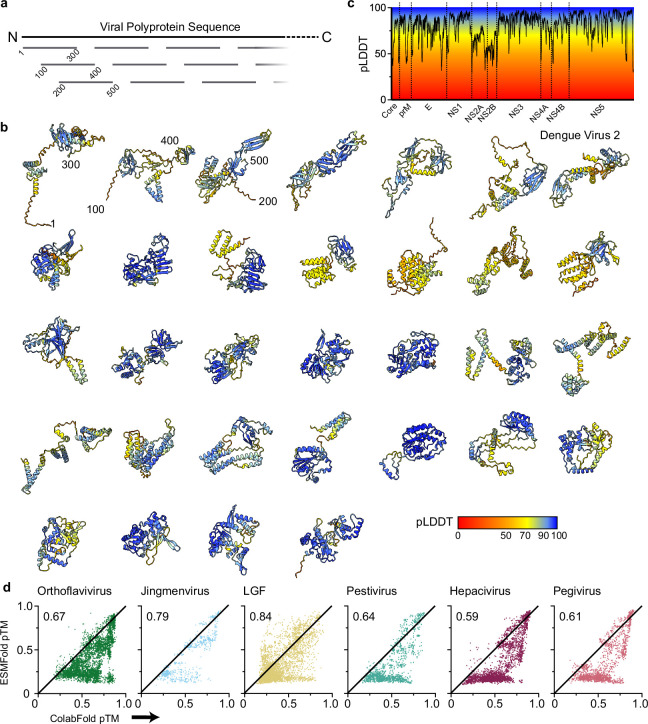
Structure prediction performance. **a**, For structural inference, all *Flaviviridae* polyprotein sequences were split into blocks of 300 residues, each overlapping by 100 residues (461 species, 16,463 blocks in total). Residue numbers are provided for the first three blocks. **b**, Representative ColabFold protein structure predictions spanning the entire Dengue Virus 2 (DENV-2) polyprotein. Residue numbers are provided as in a. Structures are colour-coded by prediction confidence scores (pLDDT), as denoted in the key. **c**, pLDDT confidence scores along the length of the DENV-2 polyprotein. Dotted lines delineate the mature proteins, which are labelled on the x-axis. **d**, Scatter plots representing predicted TM-score (pTM) confidence metric for ColabFold and ESMFold for each sequence block in each genus/subclade. Numerical values provide the performance ratio between either protein structure prediction method; values below 1 indicates better performance by ColabFold.

As protein structural prediction has yet to be systematically applied in virology, we first evaluated folding performance. ColabFold performed extremely well for many virus species (for example, dengue virus 2; Fig. 1b and Extended Data Fig. 2a–c). However, its accuracy is directly proportional to the depth of the MSAs that guide structural inference^20^, with shallow MSAs producing low confidence predictions (Fig. 1c). This becomes particularly problematic for the LGF, which are poorly sampled and consequently underrepresented in sequence databases, resulting in consistently shallow MSAs (Fig. 1d).

Structural inference by ESMFold is driven by a protein language model and does not require MSAs, but is less accurate than ColabFold. A comparison of folding confidence demonstrated that ColabFold consistently outperformed ESMFold across the three major *Flaviviridae* clades (Fig. 1e and Extended Data Fig. 2d). However, for the LGF, ESMFold yields informative predictions from sequences for which ColabFold fails, and this proved important for downstream analysis.

## *Flaviviridae* glycoprotein discovery

Orthoflaviviruses, including yellow fever virus (the canonical species for the group), tick-borne encephalitis virus (TBEV) and dengue virus (DENV), are predominantly vector-borne, although exceptions exist^21^. These viruses possess the E glycoprotein, a prototypical class II fusion protein. Structurally and functionally homologous class II fusion proteins have been identified both in viruses (for example, Gc in the bunyaviruses) and in eukaryotes (for example, HAP2 in plants, protists and invertebrates) and they are expected to share a common ancestor^22–26^. In viruses, class II fusion proteins are accompanied by a partner glycoprotein that is responsible for regulation and/or chaperoning of the fusogenic component. In the orthoflaviviruses this function is performed by the small glycoprotein prM^27^.

Identifying membrane fusion mechanisms in the hepaciviruses, pegiviruses and pestiviruses has proved more challenging. These viruses possess E1 and E2 glycoproteins that work in concert to achieve pH-dependent membrane fusion. The sequences of E1E2 bear no similarity to prM/E, and experimental structures of E2 from prototypical hepaciviruses and pestiviruses reveals folds that are broadly dissimilar from one another and from the E glycoprotein in orthoflaviviruses^28–32^. Recent cryo-electron microscopy analyses suggest that E1 adopts a unique fold, unlike that of any other known protein^33,34^.

Whether E1E2 represents a novel and as yet uncharacterized fusion mechanism or a highly divergent iteration of a class II system remains to be determined. Understanding the distribution and characteristics of glycoproteins across the *Flaviviridae* would likely provide insights on this. To achieve this we performed pairwise Foldseek^35^ structure similarity searches against the *Flaviviridae* protein foldome using a custom reference library comprising selected experimental glycoprotein structures from the Protein Data Bank (PDB) and published ColabFold models^36^.

**Fig. 2:**
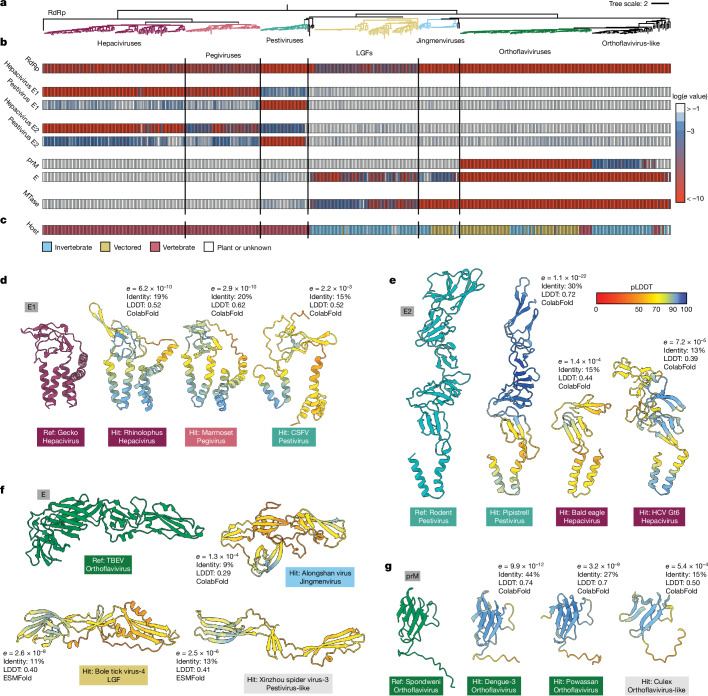
Discovery of glycoproteins across the *Flaviviridae.* **a**, RdRp phylogeny rooted on the tombusviruses (removed for visualization), with each genus or subclade colour-coded as in Fig. 1a. **b**, Foldseek structure-based homology *e*-value heat maps for the stated references, colour-coded as shown in the key. In the case of E1, E2, E, prM and MTase the values represent summary *e* values after comparison with a range of relevant reference structures (Methods). **c**, Host species tropism for each virus. ‘Vectored’ refers to those assigned as ‘Yes’ or ‘Potentially’ in Supplementary Table 3. Vertical lines within the heat map demark divisions between major clades. **d**–**g**, Representative reference structures and Foldseek hits for E1 (**d**), E2 (**e**), E (**f**) and prM (**g**). For each hit only the Foldseek-aligned residues are shown for any given structure, metrics provide *e* value, sequence identity, structural alignment score (local distance difference test (LDDT), ranging from 0 to 1) and protein structure prediction method. Predicted structures are colour-coded by pLDDT confidence scores, as shown in the key. In **d**,**e**, the reference structures are previously published ColabFold models^36^, In **f**,**g**, experimental structures are used (PDB: 7QRF and 6ZQI, respectively). CSFV, classical swine fever virus; HCV, hepatitis C virus.

**Extended Data Fig. 3:**
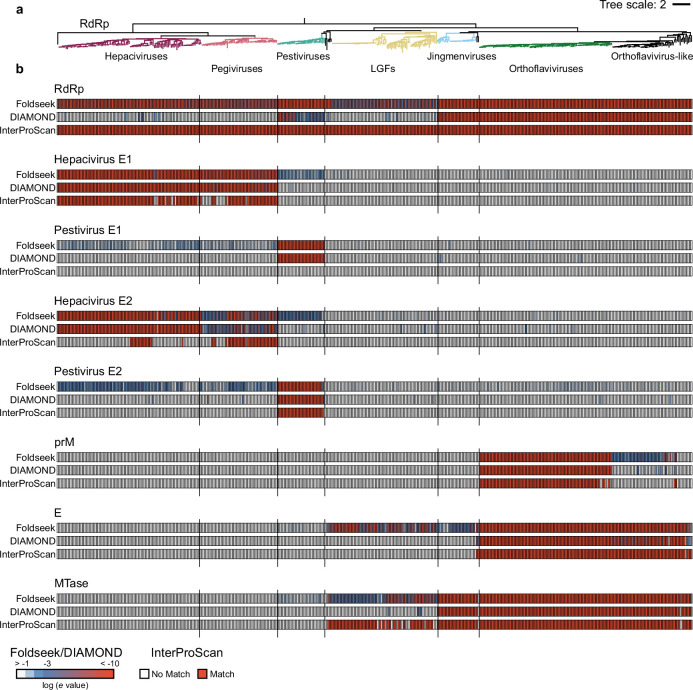
Benchmarking with sequence-based homology searches. **a**, RdRp phylogeny, as in Fig. 2a. **b**, Heatmap comparison of homology detection using Foldseek (as in Fig. 2), DIAMOND and InterProScan for the stated reference proteins. DIAMOND results represent a pure sequence-based recapitulation of the Foldseek search (i.e., all query and reference structures used in Foldseek were represented by their cognate protein sequences for DIAMOND analysis). Foldseek and DIAMOND data are log e-values and are colour-coded as in the key. InterProScan results provide a simple binary score: match (red) or no match (white). Vertical lines demark divisions between major clades.

To benchmark our approach we performed parallel analyses using state-of-the-art sequence-based approaches (DIAMOND and InterProScan^37,38^), which did not detect deep homology, even for highly conserved targets (Extended Data Fig. 3). By contrast, Foldseek demonstrated strong sensitivity, successfully detecting unambiguous structural homology between Pegivirus/Hepacivirus and Pestivirus E1 even though they share only 10–15% amino acid sequence identity (Fig. 2b,d). Relative to E1, the Pegivirus/Hepacivirus and Pestivirus E2 are structurally divergent^36^, nonetheless, Foldseek identified reciprocal structural similarity focussed on the C-terminal portion of E2 where sequence identity ranged from 8.5 to 15% (Fig. 2b,e). The distribution of E1 and E2 were in near-perfect correlation, consistent with mechanistic interdependence, and we found no evidence for E1E2-like folds outside of Pegivirus/Hepacivirus and Pestivirus groups.

**Extended Data Fig. 4:**
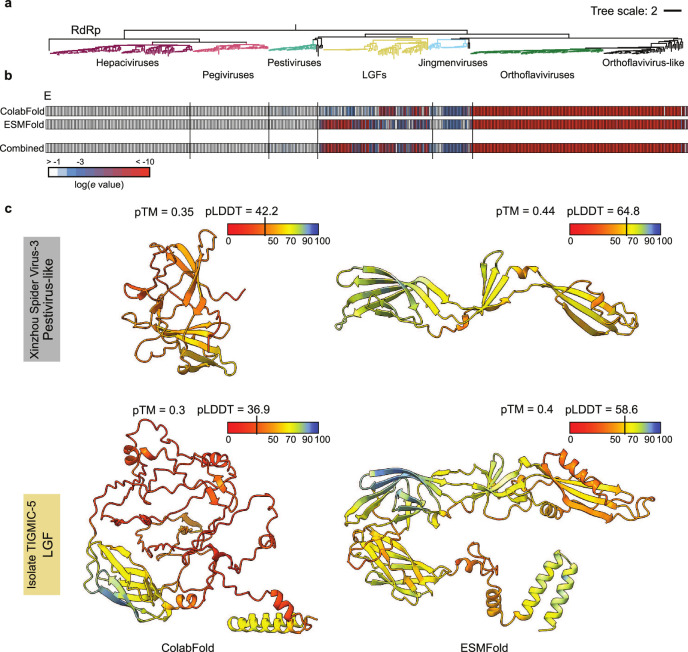
ESMFold permits unambiguous detection of glycoproteins in divergent species. **a**, RdRp phylogeny, as in Fig. 2a. **b**, E glycoprotein Foldseek e-value heatmaps for *Flaviviridae* structures predicted only by ColabFold (top), only by ESMFold (middle) or when combined (bottom). **c**, Representative examples of targets that are predicted well by ESMFold, but poorly by ColabFold. Structures are colour-coded by pLDDT confidence scores, as shown in the key. pLDDT and pTM metrics are provided for each model.

**Extended Data Fig. 5:**
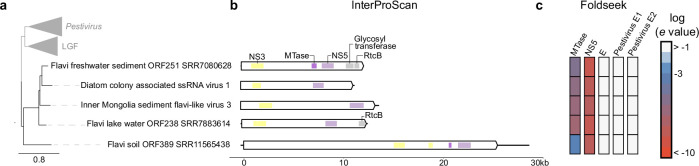
Analysis of environmental pesti-like viruses. **a**, Large genome flavivirus/*Pestivirus* subset of the *Flaviviridae* phylogeny (Tree 18) collapsed to highlight the environmental pesti-like viruses for which no glycoproteins were identified. The scale bar denotes the number of amino acid substitutions per site. **b**, Genome organisation is provided for each species, with annotations based on conserved domain sequence searches. **c**, Foldseek e-value heatmaps for the indicated reference proteins, values are log transformed and colour-coded as shown in the key. For E, E1 and E2 the values represent summary e-values after comparison with a range of relevant reference structures, as described in the methods.

We mapped structural homologues of E glycoprotein to the orthoflaviviruses, orthoflavivirus-like, jingmenviruses, LGF and pestivirus-like species that sit basal to the classical Pestivirus genus (for example, Xinzhou spider virus 3) (Fig. 2b,f). For the most divergent sequences (for example, LGF and pestivirus-like viruses) detection required ESMFold structures, emphasizing the value of using complementary prediction methods (Extended Data Fig. 4). A notable exception was a group of viruses of unknown hosts discovered in environmental samples^39–41^ (for example, Inner Mongolia sediment flavi-like virus 3) for which no glycoproteins were identified (Extended Data Fig. 5). Whether these represent species without structural proteins or partial genomes remains to be determined.

**Extended Data Fig. 6:**
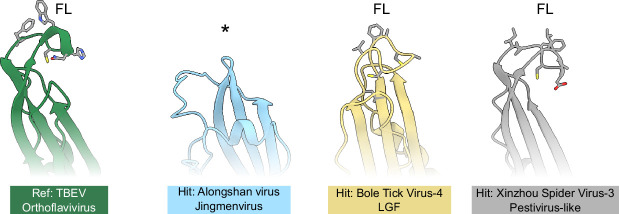
Absence of a fusion loop in E glycoprotein homologues of the jingmenviruses. Structurally conserved E protein fusion loops (FL) were found in orthoflavi-, LGF-, and in pesti-like viruses. The FL is absent from the E protein homologue of the jingmenviruses (its expected location is marked by an asterisk). Amino acid side chains are shown for the FL only.

For most E glycoprotein homologues, the predicted structures were sufficient to identify the hydrophobic fusion loop at the tip of domain II, which inserts into host membranes and is central to the class II fusion mechanism (Extended Data Fig. 6). However, the fusion loop was absent from the jingmenvirus E homologues, suggesting substantial mechanistic divergence in these viruses. We were only able to detect the prM partner glycoprotein within the orthoflaviviruses and some orthoflavivirus-like viruses (Fig. 2b,g). A critical function of prM is occluding the fusion loop of E during particle maturation and we may yet expect to find orthologous partners in other clades.

## Glycoproteins follow ecological niche

**Extended Data Fig. 7:**
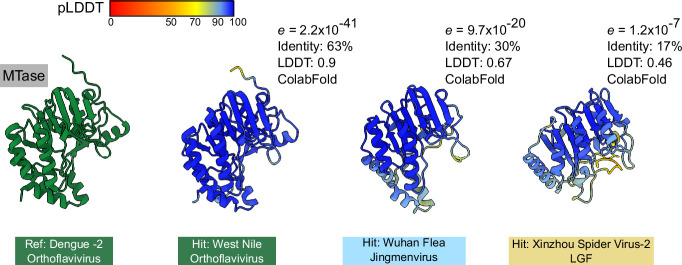
Foldseek detection of methyltransferase in diverse viruses. DENV-2 reference structure and Foldseek hits for MTase. For each hit, only the Foldseek-aligned residues are shown, metrics provide e-value, sequence identity (%), structural alignment score (LDDT, ranging from 0 to 1), and protein structure prediction method. Predicted structures are colour-coded by pLDDT confidence scores, as shown in the key.

We could assign either E1E2 or E glycoproteins to the vast majority of species in the *Flaviviridae* (Fig. 2b). Their distributions divide the family broadly in two, although this division is incongruent with the RdRp phylogeny, suggesting a complex evolutionary history. In particular, the pestiviruses and LGF, which represent sister clades on the RdRp tree, possess E1E2 and E, respectively. Although mapping glycoproteins was the primary focus of our study, we also compared the foldome against other *Flaviviridae* proteins from the PDB. In doing so we observed that all species with a methyltransferase (MTase) also possess an E glycoprotein homologue, and all species with E1E2 (that is, the hepaciviruses, pegiviruses and pestiviruses) lack MTase (Fig. 2b and Extended Data Fig. 7). Viruses with MTase undergo cap-dependent translation, whereas those without MTase rely on an internal ribosome entry site^42^ (IRES). We reasoned that E–MTase and E1E2–IRES may represent divergent co-adaptations to particular ecological niches, so we compared virus–host associations across the phylogeny (Fig. 2c). Viruses with E–MTase infect a variety of hosts, including those that are transmitted between vertebrates by invertebrate vectors (for example, DENV by *Aedes* mosquitoes). By contrast, E1E2–IRES was strictly correlated with vertebrate hosts. This suggests that the gain of E1E2 and an IRES, with the concomitant loss of E and MTase, represent a molecular commitment to the vertebrate niche. Moreover, on the basis of the underlying RdRp phylogeny, this commitment to vertebrate infection is likely to have occurred twice in the *Flaviviridae*, once for Pegivirus/Hepacivirus and once for the pestiviruses.

## LGFs harbour novel and acquired proteins

Our structure-guided approach can offer new insights into divergent and/or poorly characterized viruses such as those found in the LGF. Whereas the majority of LGF species are likely to infect invertebrates, there is evidence that one subclade—the bole tick virus group—are capable of tick-borne infection of mammals, including humans^6^. This group may represent an emergent threat to public health and warrants closer scrutiny.

**Fig. 3:**
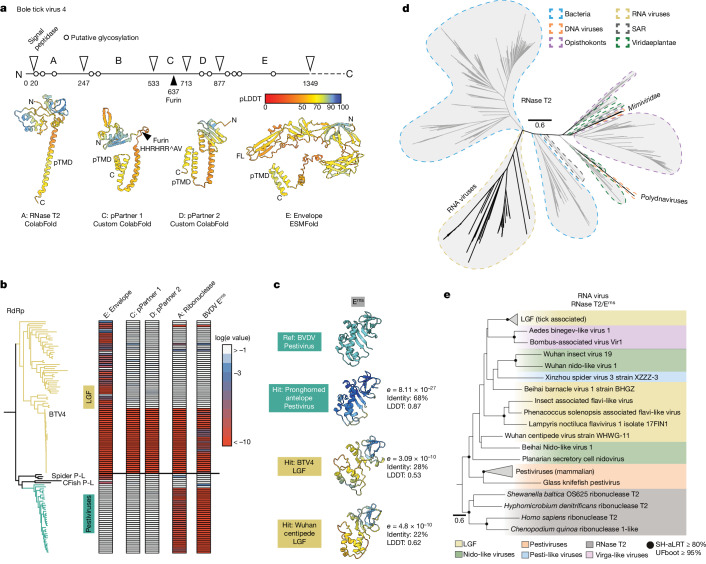
Novel and acquired proteins in a large genome flavivirus. **a**, N-terminal glycoproteins from BTV4. Linear representation of the BTV4 polyprotein displays location of putative glycosylation and the predicted signal peptidase cleavage sites that delineate five mature proteins (labelled A–E). For each of these mature proteins, the highest confidence models are shown from three prediction methods (ColabFold, ColabFold with custom MSAs and ESMFold). Protein B only yielded low-confidence models and is not shown. Each protein contains a putative transmembrane domain (pTMD), protein C contains a canonical furin cleavage site, and the conserved fusion loop (FL) of E is also annotated. **b**, LGF/Pestivirus lineage RdRp phylogeny and Foldseek *e*-value heat maps for the stated reference structures. Annotations provide the location of BTV4 (reference), the spider pestivirus-like viruses (Spider P-L), and the cartilaginous fish pestivirus-like viruses (CFish P-L). **c**, Example Foldseek hits against an experimental structure of bovine viral diarrhoea virus E^rns^ ribonuclease (PDB: 4DVK). BVDV, bovine viral diarrhoea virus. **d**, Ribonuclease T2 (RNase T2) sequence phylogeny, with domains of life and viruses colour-coded as shown in the key. The scale bar indicates phylogenetic distance as number of substitutions per site. This protein has been independently acquired once by RNA viruses and twice by DNA viruses (in *Mimiviridae* and polydnaviruses). The RNA virus clade is nested within bacterial instances of the RNase T2, suggesting a single horizontal gene transfer event. **e**, Phylogeny of the RNA virus RNase T2/E^rns^ clade rooted on non-viral sequences, with viral clades colour-coded as shown in the key. An uncollapsed version containing all tip labels is provided in Supplementary Fig. 8.

Focusing on Bole tick virus 4 (BTV4), we examined the N-terminal portion of the polyprotein proximal to the E glycoprotein homologue. The N-terminal structural proteins of *Flaviviridae* polyproteins are typically processed by host signal peptidases to liberate the mature proteins. We used cleavage site prediction to identify five putative protein coding sequences (labelled A–E; Fig. 3a). Protein structures were predicted using three approaches—ESMFold, ColabFold and ColabFold with manually curated MSAs (Methods). For each sequence, we provide the highest confidence model produced by any of these methods (Fig. 3a).

The largest and most C-terminal protein is the E glycoprotein homologue. ESMFold produces a model in which the transmembrane domain and fusion loop are juxtaposed; this is consistent with experimental structures of the post-fusion conformation of E^43^. Directly upstream of the E homologue are two smaller proteins for which custom ColabFold yielded the optimal predictions. Although neither protein shares direct homology with prM, they both have a similar organization, consisting of a small globular domain anchored by a putative transmembrane domain. Given their direct proximity to the E homologue, we suggest that these are partner proteins that provide chaperoning to the class II fusogen. Indeed, the putative partner 1 possesses a furin cleavage site, analogous to prM, that would enable proteolysis during secretion, akin to the maturation of Orthoflavivirus particles^27^. Protein coding sequence B yielded low confidence predictions from each folding approach (not shown). The most N-terminal sequence was identified as a T2 family ribonuclease (RNase T2), with homologues across the tree of cellular life^44^.

We used Foldseek to investigate the distribution of these protein structures across the LGF/Pestivirus clade (Fig. 3b). Homologues of BTV4 E glycoprotein were detected throughout the LGF and in pestivirus-like viruses identified in spiders and cartilaginous fish that fall basal to members of the classical genus Pestivirus. Therefore, using a proximal reference (BTV4 E glycoprotein), we provide evidence for the loss of E and gain of E1E2 at the genesis of the pestiviruses. By contrast, the putative partner proteins were confined to the Bole tick virus subclade, and structural similarity searches against current protein databases (for example, PDB and AlphaFoldDB) revealed no homologues. Therefore, these proteins are likely adaptive features, specific to these viruses.

BTV4 RNase T2 has homologues throughout the Bole tick virus subclade and, notably, across the genus Pestivirus, where the homology maps to the E^rns^ ribonuclease. Foldseek searches against pestivirus E^rns^ revealed a reciprocal distribution of homology (Fig. 3b,c). Phylogenetically, the LGF/pestivirus E^rns^ form a deep branch amongst homologous RNase T2 sequences from viruses, bacteria, plants and animals (Fig. 3d,e). Together, this indicates that E^rns^ originated in a distant ancestor of the pestiviruses and LGFs, probably from a single horizontal gene transfer of a bacterial RNase T2. Moreover, the distribution of E^rns^ is broadly concordant with the RdRp phylogeny, suggesting that E^rns^ has been continuously retained in certain species and lost in others (Fig. 3e), rather than undergoing genetic exchange within the clade. Further instances of RNase T2 from nidovirus-like and virgavirus-like viruses were also nested within the E^rns^ tree, indicating onward genetic transfer to other RNA viruses.

## Evolutionary history of the *Flaviviridae*

**Fig. 4:**
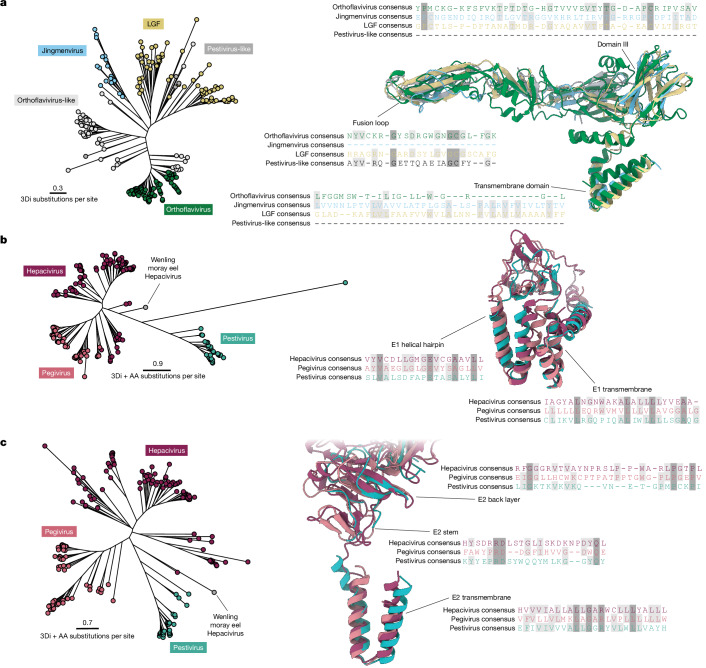
Structurally informed phylogenetics. **a**, Left, 3Di-based E structural phylogeny. The scale bar indicates the number of 3Di character substitutions per site (see Methods for details of tree selection). Right, representative structures superposed using flexible FATCAT^49^ with a ColabFold model of West Nile virus E protein as reference (green). Structures are colour-coded as in the phylogeny. The protein alignments provide structurally aligned consensus-level amino acid sequences for the fusion loop, domain III and transmembrane domain. Conserved residues are highlighted. **b**, Left, combined 3Di and amino acid-based E1 structural phylogeny. The scale bar indicates the number of 3Di and amino acid character substitutions per site. Right, representative structures are superposed with Hepacivirus F E1 protein. Alignments demonstrate consensus-level homology in the E1 helical hairpin and transmembrane domain. Structures are colour-coded as in the phylogeny. **c**, Left, combined 3Di and amino acid-based structural phylogeny of E2 protein. Right, representative structures are superposed with Hepacivirus F E2. Consensus-level homology in E2 back layer, stem and transmembrane domain are provided. The basal Wenling moray eel Hepacivirus is marked in both the E1 and E2 trees. AA, amino acid.

**Extended Data Fig. 8:**
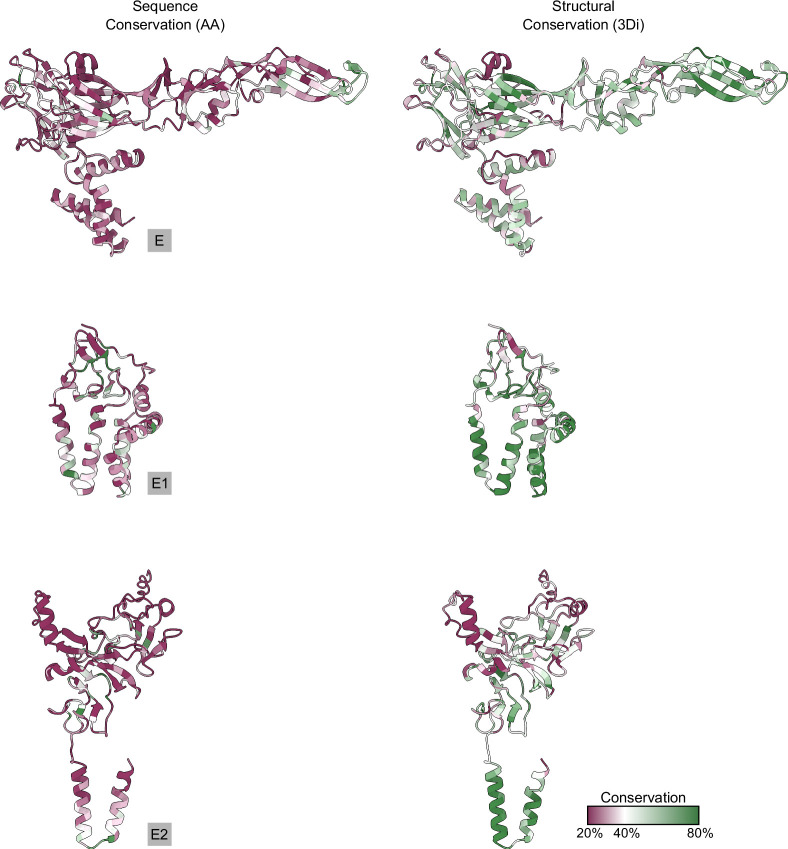
Conservation of structure revealed through the Foldseek 3Di alphabet. Representative structures of E (West Nile virus) or E1 and E2 (Hepacivirus F) colour-coded by either sequence or structural conservation, as denoted in the key. In both cases values represent percentage conservation of the consensus for each structurally aligned protein (see Methods for details), with amino acid residues representing protein sequence and the 3Di structural alphabet representing protein structure.

**Extended Data Fig. 9:**
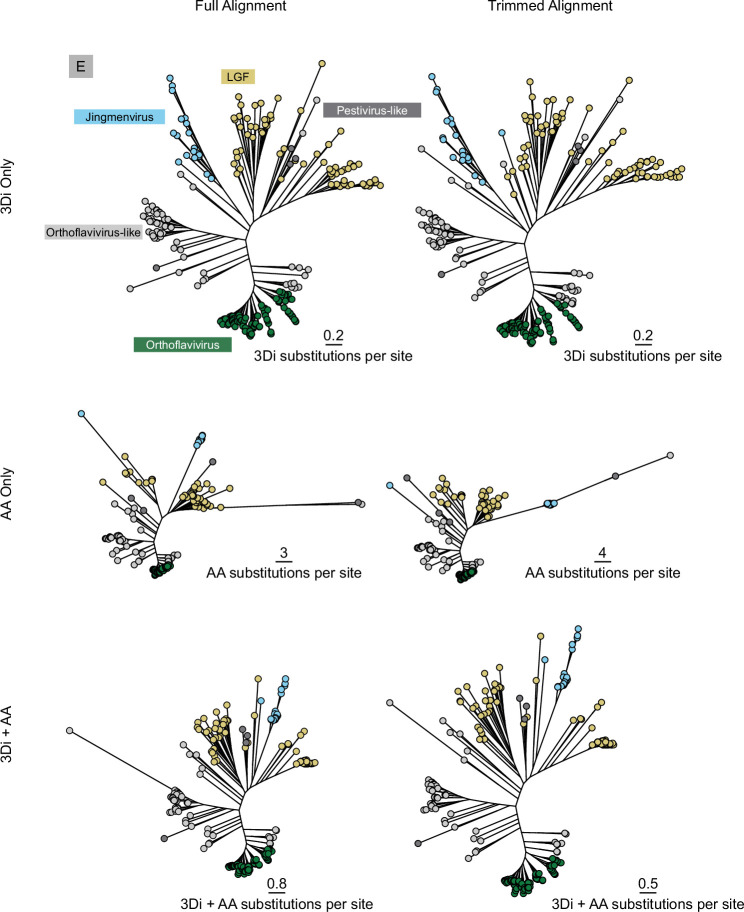
Structurally aligned E glycoprotein phylogenies. Protein sequences were aligned using their 3Di structural representation (see Methods for details). Phylogenies were reconstructed using 3Di sequence alone (top), amino acid (AA) sequence alone (middle) or combined 3Di and AA sequence. Right hand trees are derived from alignments trimmed with a gap threshold of 35%. Scale bars indicate substitutions per site for either the 3Di, AA or combined sequences, respectively. Tip shapes are colour-coded by genus/subclade as in Fig. 1a. All phylogenetic trees are provided in the associated Zenodo repository.

**Extended Data Fig. 10:**
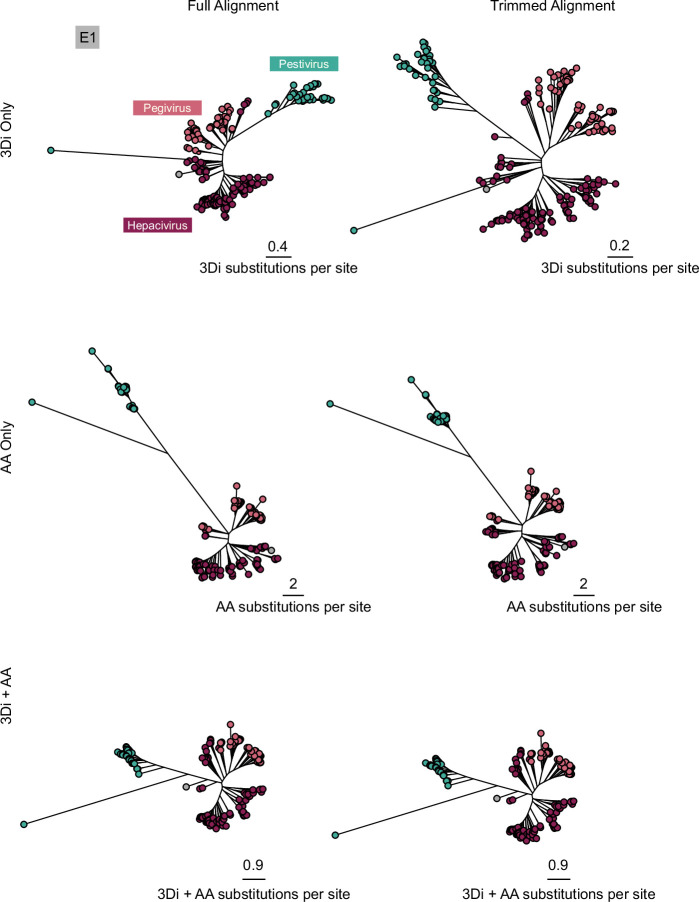
Structurally aligned E1 glycoprotein phylogenies. Protein sequences were aligned using their 3Di structural representation (see Methods for details). Phylogenies were reconstructed using 3Di sequence alone (top), amino acid (AA) sequence alone (middle) or combined 3Di and AA sequence. Right hand trees are derived from alignments trimmed with a gap threshold of 35%. Scale bars indicate substitutions per site for either the 3Di, AA or combined sequences, respectively. Tip shapes are colour-coded by genus/subclade as in Fig. 1a. All phylogenetic trees are provided in the associated Zenodo repository.

**Extended Data Fig. 11:**
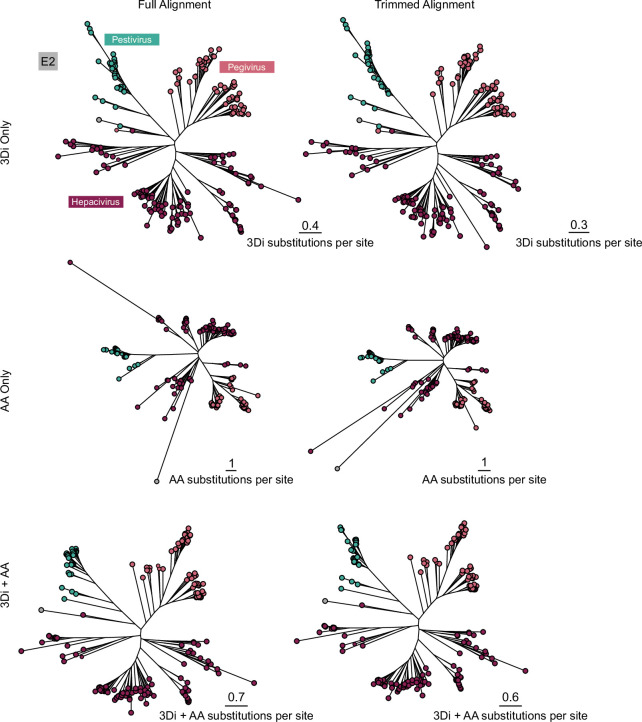
Structurally aligned E2 glycoprotein phylogenies. Protein sequences were aligned using their 3Di structural representation (see Methods for details). Phylogenies were reconstructed using 3Di sequence alone (top), amino acid (AA) sequence alone (middle) or combined 3Di and AA sequence. Right hand trees are derived from alignments trimmed with a gap threshold of 35%. Scale bars indicate substitutions per site for either the 3Di, AA or combined sequences, respectively. Tip shapes are colour-coded by genus/subclade as in Fig. 1a. All phylogenetic trees are provided in the associated Zenodo repository.

Our approach enabled the discovery of glycoproteins (and other features) across the entire *Flaviviridae*. To better understand the evolutionary events that gave rise to this distribution of molecular characteristics we utilized a method^45^ that leverages structural conservation (as represented in the Foldseek 3Di alphabet; Extended Data Fig. 8) to guide and augment traditional amino acid-based evolutionary analyses (Methods). This revealed consensus-level glycoprotein sequence similarities, indicative of shared ancestries, that can be estimated through phylogenetic modelling (Fig. 4a–c and Extended Data Fig. 9–11).

The optimal E glycoprotein phylogeny largely reflects the RdRp tree, with E homologues from orthoflaviviruses, orthoflavirus-like viruses jingmenviruses and LGFs distributed across various subclades (Fig. 4a). Notably, the E protein homologues in pestivirus-like viruses of spiders fell within the LGF glycoprotein clade, similar to the RdRp tree topology (Fig. 3b). This again suggests that the acquisition of E1E2, accompanied by loss of E, was a defining event in the emergence of the pestiviruses from an LGF-like progenitor.

Both the E1 and E2 phylogenies indicate a common glycoprotein ancestry in Pegivirus/Hepacivirus and Pestivirus groups (Fig. 4b,c), even though they are paraphyletic in the RdRp phylogeny (Fig. 1a). Of note, the Wenling moray eel hepacivirus (which is basal to the pegivirus/hepacivirus RdRp lineage) sits at the intersection of the Pegivirus/Hepacivirus and Pestivirus E1 and E2 clades, consistent with deep ancestry. We could not detect any significant structural homology between E1E2 and E (Fig. 2), or identify intermediate forms between these glycoprotein systems, further suggesting they are mechanistically distinct. We therefore propose that E1E2 represents a novel class of fusion protein. Moreover, the structural and sequence conservation within E1 and the basal portion of E2 (Figs. 2 and 4b,c and Extended Data Fig. 8) suggests a mechanistic role requiring experimental exploration.

**Fig. 5:**
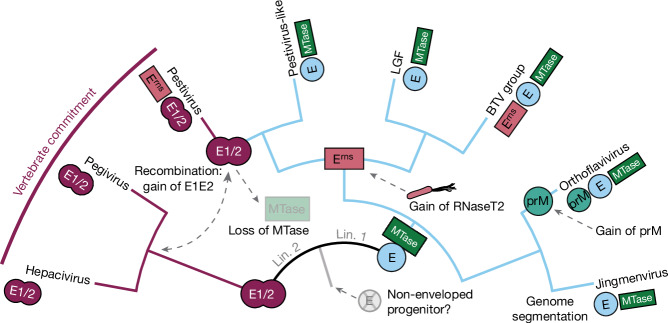
Proposed evolutionary history of the *Flaviviridae.* Illustrative cladogram showing the key protein acquisition and loss events across the major *Flaviviridae* clades. The two major lineages are labelled (Lin. 1 and Lin. 2), near the root. Each clade, displayed as a tip, is annotated with symbols representing the presence of key proteins. Branches are highlighted to denote the lineage-specific presence of envelope protein E (in light blue) or E1/E2 (in maroon). Major nodes are emphasized with larger symbols to infer the ancestral emergence of each protein within the *Flaviviridae*. Dashed lines and arrows denote the loss or gain of specific proteins, highlighting potential recombination events and gene transfers. Image of *Aquifex pyrophilus* by Guillaume Dera; CC BY 1.0 (https://creativecommons.org/licenses/by/1.0/).

On the basis of our combined analyses, we propose a *Flaviviridae* evolutionary history shaped by gains and losses of defining protein functions, as summarized in Fig. 5. The most parsimonious interpretation of the data is that Orthoflavivirus/jingmenvirus and LGF/Pestivirus clades (lineage 1) arose from an ancestor that possessed the E glycoprotein and performed cap-dependent translation, necessitating MTase. By contrast, the Pegivirus/Hepacivirus clade (lineage 2) arose from an ancestor that possessed E1E2 glycoproteins and lacked MTase, implying reliance on IRES-dependent translation.

Compared with lineage 2 (where the hepaciviruses and pegiviruses share relatively conserved RdRp and glycoproteins), lineage 1 has undergone extensive diversification. Genome segmentation occurred in the jingmenviruses, with coincident divergence in their E glycoprotein^16^, including the apparent loss of its canonical fusion loop. The orthoflaviviruses gained prM, a partner to the E glycoprotein, probably derived from a host chaperonin^46^. A sister lineage gives rise to the LGF and Pestivirus clades, in which an ancestral species gained RNase T2 from bacteria. Whereas the LGF, including basal pesti-like viruses, possess E glycoprotein, all pestiviruses possess E1E2 glycoproteins, homologous to those found in the hepaciviruses and pegiviruses. This indicates a switch in glycoprotein systems through an inter-genus horizontal gene transfer; with concomitant loss of MTase. In lineages one and two, the presence of E1E2 (and IRES-dependent translation) is strictly associated with viruses of vertebrates, suggesting a molecular commitment to ecological niche.

The characteristics of the common ancestor of the entire *Flaviviridae* remain speculative and, on the basis of the taxonomic distribution of infected hosts and the existence of endogenous viral elements, may have originated over 900 million years ago^47^. This ancestor possibly contained core NS3 and NS5 proteins and potentially an MTase. Although the absence of RrmJ-like methyltransferases (as found in the *Flaviviridae)* in other members of the phylum Kitrinoviricota^48^, hints that MTase might have been acquired at the base of lineage 1. It remains unclear whether this progenitor possessed an envelope, therefore necessitating fusion glycoproteins; although it is noteworthy that, with the exception of *Togaviridae*, *Matonaviridae* and *Flaviviridae*, all Kitrinoviricota families (*n* = 21) are non-enveloped.

Ultimately, the origins of the E and E1E2 glycoproteins remain uncertain. We cannot exclude the possibility of hidden E glycoprotein horizontal gene transfer within the *Flaviviridae*. For instance, alternative phylogenetic models to the one in Fig. 4a place the jingmenvirus E within the LGF clade (although this may be an artefact of long branch attraction; see Methods and Extended Data Fig. 9). Moreover, we have observed apparent ‘genetic piracy’ by LGFs (for example, RNase T2 (Fig. 3) and in the recent work of Petrone et al.^13^) and therefore these viruses may have acquired their glycoproteins by horizontal gene transfer from within the *Flaviviridae* or beyond. Resolving these questions will probably require the discovery of further novel species and the inclusion of diverse taxa from beyond the *Flaviviridae*, such as the *Matonaviridae*, *Togaviridae* and *Peribunyaviridae*, which also possess class II fusion proteins. However, these analyses, across a wide diversity of the RNA virosphere, are likely to challenge even the highly sensitive structure-driven approaches outlined here.

## Discussion

The limited ability of sequence-based methods to detect deep homology has resulted in significant ambiguity regarding the distribution and classification of fusion glycoproteins across the *Flaviviridae*. Our work, using protein structure prediction, has discovered previously unknown glycoproteins in more than 100 species and reveal unambiguous structural and sequence similarity between E1E2 in the hepaciviruses, pegiviruses and pestiviruses, indicative of inter-genus genetic exchange. The absence of homology between the E glycoprotein (class II fusion mechanism) and E1E2, even in basal species, provides the strongest evidence yet of a novel fusion mechanism in the Pegivirus/Hepacivirus and Pestivirus groups. Through comparison to host tropism, we found that E1E2 is strictly correlated with infection of vertebrates, suggesting a molecular commitment to virological niche.

Beyond biological insights, our work demonstrates that protein structure prediction and structure-guided homology searches outperform the gold standard sequence-based approaches to provide unprecedented clarity to the evolution of viruses. Whereas AlphaFold-based methods offer unparalleled accuracy^20^, protein language model-based systems such as ESMFold may be more capable of exploring the ‘viral dark matter’ revealed by metatranscriptomics. In sum, our study offers a new state-of-the-art approach for understanding the diversity and distribution of protein functions throughout the virosphere.

## Methods

### Compilation of *Flaviviridae* sequence set

#### Retrieval of flavivirus genomes

Flavivirus sequences were collected using the search phrase “*Flaviviridae* taxid 11050 and Unclassified *Flaviviridae* taxid 38144” in the NCBI Virus Database on 15 December 2022. The search was complemented by referencing sequences from Mifsud et al.^50^ and supplemented with sequences from the NCBI nucleotide database using the search phrase “flavi[All Fields] OR pesti[All Fields] OR hepaci[All Fields] OR pegi[All Fields] AND viruses[filter]” on the same date. Additional sequences were later retrieved from publications that had sequences not available in GenBank at the time^12,40,51–56^.

#### Sequence set curation

Sequences were clustered to a 95% nucleotide identity threshold to approximate a species-level distinction, excluding the LGF tick-associated clade. Clustering was performed using CD-HIT (v4.6.1)^57^ with non-default parameters “cd-hit-est -c 0.95 -n 9”. Subsequently, the clustered sequence set was manually curated by removing incomplete coding regions. Sequences shorter than 2,000 nucleotides in length were removed, with the exception of the jingmenviruses where segments are known to be <2000 nucleotides in length. These nucleotide sequences were translated using the Geneious Prime Find ORFs tool (v2022.0) (https://www.geneious.com/)^58^ and along with protein sequences aligned to annotated reference sequences (where available) using MAFFT FFT-NS-I X2 (v7.402) to assess genome completeness^59^. This was complemented by predicting conserved domains using the InterProScan software package (v5.56-89.0) with the SFLD (v4.0), PANTHER (v17.0), SuperFamily (v1.75), PROSITE (v2022_01), CDD (v3.18), Pfam (v34.0), SMART (v7.1), PRINTS (v42.0), and CATH-Gene3D databases (v4.3.0)^38^. Sequences determined to contain partial coding sequences were removed from the subsequent analyses.

#### Discovery of novel LGF sequences

Tick-associated LGFs are of particular interest due to the recently reported association between Haseki tick virus and tick-borne infectious disease in humans^6^. To identify related viruses, we screened the Sequence Read Archive (SRA) RdRp microassemblies generated by Serratus^60^ using DIAMOND BLASTx (v2.0.9)^37^ (*e*-value threshold of 10^−5^ and the “--ultra-sensitive” flag)^37^ with Haseki tick virus (UTQ11742) as the query. An *e*-value threshold of 1.6 × 10^−15^ was established to restrict the number of libraries for reassembly to a manageable quantity. This threshold was determined based on the organism associated with the SRA library and the percent identity values. The 319 SRA libraries that meet this threshold were processed following the BatchArtemisSRAMiner pipeline^61^. In brief, raw FASTQ files were retrieved using Kingfisher (v0.3.0) (https://github.com/wwood/kingfisher-download), quality trimming and adapter removal using Trimmomatic (v0.38)^62^ with parameters SLIDINGWINDOW:4:5, LEADING:5, TRAILING:5, and MINLEN:25 and de novo assembly using MEGAHIT (v1.2.9)^63^ with default parameters. The assembled contigs were compared to the NCBI non-redundant protein database (as of March 2023) and a custom *Flaviviridae* protein database using DIAMOND BLASTx as described above. All novel flaviviruses predicted to contain complete coding sequences identified by this method (including those outside of the LGF group) were included in phylogenetic analyses.

### Structure prediction and homology search

#### Systematic protein structure prediction

We adopted a strategy to overcome incomplete and ambiguous genome annotations, and generate sequence lengths that are amenable to rapid inference of structure. *Flaviviridae* polyprotein amino acid sequences were broken into sequential 300-residue blocks with a 100-residue overlap. However, most polyproteins are not equally divisible by 300, therefore, we set the final sequence block to cover the final 300 residues of the polyprotein, irrespective of overlap with the penultimate block. This resulted in 16,463 sequence blocks from 561 species (558 from the *Flaviviridae* and 3 from the Tombusvirus outgroup). Structures were predicted for each sequence using the ColabFold (v1.5.1) implementation of AlphaFold2 (v2.3)^19^, with default settings but only generating a single model per target, performed using Google Colab cloud computing. Structural inference was also performed with ESMFold (v1)^18^ (using the 3 billion parameter ESM-2 model), on local compute (Nvidia V100 GPU + 32GB vRAM). This resulted in a total of 32,926 structural models. Custom Python scripts were used to break up sequences for folding and extract metrics from outputs (that is, pLDDT confidence and MSA depth). For inference of putative mature protein sequences (Fig. 3) the SignalP server (v6.0) was used to predict the junctions between viral proteins^64^. For custom ColabFold inference (Fig. 4), whole polyprotein sequences of the Bole tick virus group were aligned using MAFFT, MUSCLE (v5.1)^65^, and subalignments covering only the putative protein sequences were converted to the.a3m format and used as input for ColabFold structure prediction^19^. All predicted structures and summary statistics are included in the associated Zenodo repository (10.5281/zenodo.11092288)^66^. Representative structural superpositions (Fig. 4) were performed using FATCAT (v2.0)^49^. All structural visualizations were prepared for publication using UCSF ChimeraX^67^.

#### Structural homology searches

We used Foldseek in exhaustive search mode to cross compare the *Flaviviridae* protein foldome with a library of reference structures drawn from the protein database and ColabFold models of particular targets (see below). Foldseek was set to output *e* values, structurally aligned amino acid sequences, % identity of aligned residues, bit score and lddt structural similarity, with an *e*-value cut-off of 0.1 to eliminate low probability hits and reduce the size of the output datafile. To interrogate the output data, the lowest *e*-value scores for any given species against any given reference structure were extracted using a custom python script. Where multiple references were used for a single protein the lowest *e* value against any given species was chosen. This data was plotted against sequence-based phylogenies using the Interactive Tree Of Life^68^. Representative hits (Fig. 2d–g and Extended Data Fig. 7) were selected manually to reflect the levels of similarity and divergence in structure and sequence. All reference structures are included in the underlying data, the following experimental structures were used from the PDB: 6ZQI (Spondweni virus E and prM), 1L9K (DENV-2 MTase), 5F3Z (DENV-3 RdRp), 7QRF (TBEV E and prM), 7V1E (Omsk haemorrhagic fever virus MTase), 7T6X (HCV E1 and E2), 6VYB (SARS-CoV-2 spike, negative control), 2YQ2 (BVDV E2) and 4DVK (BVDV E^rns^)^28,33,46,69–74^. To increase reference coverage, additional ColabFold models of DENV-1 prM E and diverse Hepacivirus, Pegivirus and Pestivirus E1 and E2 (from Oliver et al.^36^) were also used. ColabFold or ESMFold structures of BTV4 proteins (Fig. 3a) were used in downstream Foldseek analysis and as references in the assembly of continuous glycoprotein structures for the structural phylogeny work presented in Fig. 4 (see below).

#### Sequence homology search benchmarking

To demonstrate the increased sensitivity achieved through structure prediction approaches, we conducted two benchmarking analyses. In the first analysis we recapitulated the Foldseek search by querying the 300-residue blocks against the cognate protein sequences underlying the reference structure database using DIAMOND BLASTp (*e*-value threshold of 0.1 and the “--ultra-sensitive” flag)^37^. We then filtered the results to select the block with the lowest *e* value for each flavivirus and reference sequence pair. The second analysis involved annotating the complete *Flaviviridae* polyprotein sequences using the InterProScan software package (v5.65-97.0) with the AntiFam (v7.0), FunFAM (4.3.0), MobiDBLite (v2.0), NCBIfam (v13.0), SFLD (v4.0), PANTHER (v18.0), SuperFamily (v1.75), CDD (v3.20), Pfam (v36.0), SMART (v9.0), PRINTS (v42.0) and PIRSF (v2023_05) databases^38^. As *e* values are specific to each InterPro database and each utilizes their own *e*-value post-processing, direct comparisons are not feasible. Consequently, as advised, all matches were considered tentative hits (https://interproscan-docs.readthedocs.io/en/latest/FAQ.html).

### Phylogenetic analysis

#### NS5b phylogeny

The evolutionary relationships among the *Flaviviridae* were inferred using maximum likelihood phylogenies derived from MSAs of the highly conserved NS5b region (which encodes the RdRp). This region was extracted from each sequence by aligning polyprotein sequence subsets according to their taxonomy and using both pre-existing and newly generated NS5b annotations from InterProScan as a guide. As alignment and trimming parameters have been shown to influence the topology of the *Flaviviridae*^75^ we compared several methods resulting in 225 phylogenies. In brief, flavivirus sequences were aligned using MAFFT, MUSCLE (v5.1)^65^ and Clustal Omega (v1.2.4)^76^ with default parameters. Ambiguously aligned regions were removed using trimAl (v1.4.1)^77^ with 8 conservation thresholds (that is, minimum percentage of alignment columns to retain): 5, 7.5, 10, 12.5, 15, 17.5, 20 and 25; and 3 gap thresholds (that is, the minimum fraction of sequences without a gap needed to keep a column): 0.7, 0.8, and 0.9—as well as the automated parameter selection method gappyout.

All maximum likelihood phylogenetic trees were estimated using IQ-TREE 2 (v2.1.0)^78^. Selection of the best-fit model of amino acid substitution was inferred for a subset of phylogenies using the ModelFinder function in IQ-TREE 2^79^. In addition to the model chosen by ModelFinder (LG + F + R10) two additional models, the Le-Gascual model (LG) and FLAVI^80^ were compared. Branch support was calculated using 1,000 bootstrap replicates with the UFBoot2 algorithm and an implementation of the SH-like approximate likelihood ratio test within IQ-TREE 2^81,82^. To root the phylogeny, three members of the *Tombusviridae* family were chosen given their remote sequence similarity to the NS5 region of the *Flaviviridae*^2,10^. Phylogenetic trees were annotated using the R packages ape (v5.6.2)^83^, phytools (v1.5-1)^84^, and ggtree (v3.3.0.9)^85^ and further edited in Adobe Illustrator. Genome diagrams were constructed using a manually curated selection of predicted functional domains and visualized using gggenomes (v0.9.8.9)^86^.

For each virus sequence, host information was pulled from the corresponding GenBank ‘host’ field using Rentrez (v1.2.3)^87^ and standardized using Taxize (v0.9.1)^88^. Vector status, defined as ‘yes’, ‘no’ or ‘potentially’, was assigned by first querying the Arbovirus Catalog (https://wwwn.cdc.gov/arbocat/). Where a taxon was identified as an ‘Arbovirus’ by the Arbovirus Catalog it was assigned ‘yes’, otherwise for those listed as ‘potential arboviruses’, ‘probable arboviruses’, or those not present in the catalogue, literature on this taxa was reviewed for evidence of vector association. Three main criteria were considered: (1) whether the virus replicated in both invertebrate and vertebrate cells; (2) the phylogenetic position of the virus—that is, is the virus in the middle of an insect-specific clade?; and (3) consensus among the literature on the possibility of the virus being vectored. The assigned vector status for each taxon and the underlying evidence for this is provided in Supplementary Table 3.

#### Evaluation of topological concordance

To determine the most robust NS5b phylogeny, alignments (pre- and post- trimming) (*n* = 225) were examined for the presence of canonical RdRp motifs, misalignments, and overall pairwise identity and length. The resultant tree topology and branch support were examined in FigTree (v1.4.4)^89^. This analysis was combined with comparisons of genome composition and to previous *Flaviviridae* phylogenies^10,47,75^ to identify the most concordant topology across the multiple parameters tested. To supplement this, the R package treespace (v1.1.4.2)^90^ was used to conduct a principal component analysis (PCA) with the goal of identifying clusters of similar trees and assessing whether the selected topology is consistent with the median topology. Accordingly, Kendall–Colijn distance was calculated for each tree and used for the PCA, with two principal components retained^91^. To identify discrete clusters of related trees, pairwise distances were mapped into four clusters using hierarchical clustering (Ward’s method)^92^. Manual and distance-based inspection revealed that the alignment method drove variation in tree topology and branch lengths between phylogenies. Specifically, tree topologies and their corresponding phylogenetic distances derived from Clustal Omega were frequently topologically discordant compared to those generated by MAFFT and MUSCLE; as such, these phylogenies were excluded and the PCA was recalculated. Geometric median trees were generated from each cluster and alignment method and used to inform the selection of the final phylogeny. This phylogeny was aligned using MUSCLE with a trimAl consensus and gap threshold value of 5 and 0.9, respectively, and based on the LG + F + R10 amino acid substitution model. We further conducted an extensive stratified MUSCLE alignment analysis to validate the robustness of our NS5b alignment and resulting phylogenies which considered variations in hidden Markov model (HMM) parameters and guide tree merge orders (Supplementary Note 1 and Supplementary Figs. 2–7).

#### RNase T2 phylogeny

To infer the evolutionary history of the RNase T2 protein, sequences were obtained from the GenBank protein database for conserved domains using the queries “taxid 238513” and “taxid 238220”, literature searches^93,94^, these were supplemented with structurally homologous protein clusters identified using the AlphaFold database Foldseek clusters server^95^.

To identify unannotated RNase T2-like sequences in virus genomes, a NCBI web protein BLAST (https://blast.ncbi.nlm.nih.gov/Blast.cgi) was used with RNase T2 sequences used as a query against the clustered non-redundant protein database (as of June 2023)^96^, using the BLOSUM45 matrix and with taxonomy limited to the group ‘Viruses’ (taxid:10239). The HMM search web server (v2.41.2)^97^ was used to identify additional viral T2 RNase-like sequences. An alignment of RNase T2 sequences was used as a query against the Reference Proteomes, UniProtKB, SwissProt and PDB databases (as of June 2023), with results again limited to ‘Viruses’ (taxid:10239). This was further repeated for the PDB, SCOPe, SMART, Pfam, UniProt-SwissProt-Viral, PHROG, COG and KOG databases using the HHpred web server^98,99^ as of April 2024 and the Uniclust30 using HHsearch (v3.3.0)^100,101^. For all methods, if new virus sequences were detected, they were manually inspected for the presence of RNase T2 motifs and, in turn, used as queries. To estimate the RNase T2 phylogeny, non-viral sequences were clustered at 80% amino acid identity using DIAMOND cluster (v.2.0.9)^102^ with default parameters and aligned with the viral sequences using MAFFT and a maximum likelihood phylogeny as described above.

#### Structure-guided glycoprotein phylogenies

We implemented the approach described previously^45^ to infer glycoprotein phylogenies based on both structural and sequence homology. Owing to the arbitrary fragmentation of protein sequences into the 300-residue blocks, our predicted structures represent overlapping truncated segments of the true glycoproteins. To generate full glycoprotein structures we filtered our Foldseek results by an *e*-value cut-off of 0.001 and selected the E, E1 or E2 reference structure that had the highest bit score value for any protein block of each query virus. This reference was then used for determining the putative coordinates of each glycoprotein in the viruses’ polyproteins, defined as the start position of the earliest block’s Foldseek hit to the reference and the end position of the latest block’s hit. This process yielded 247 E, 190 E1 and 189 E2 protein sequences, the majority of which appeared to be full length glycoproteins, but with a minority of truncated sequences likely due to low-accuracy structure prediction in the protein foldome. The structure of the full glycoprotein sequences was predicted using ColabFold and ESMFold as described above, but with five ColabFold models produced for each target. The most confident prediction based on their average pLDDT values was chosen for downstream analysis.

We modified the FAMSA alignment program^103^ to use the Foldseek 3Di character substitution matrix as described previously^45^ (https://github.com/nmatzke/3diphy). We then converted our predicted full glycoprotein structures to 3Di sequences with the Foldseek ‘structureto3didescriptor’ option and used the modified FAMSA aligner to infer structural, 3Di sequence alignments of the E, E1 and E2 protein sets. These MSAs are based on the homology between the 3Di characters corresponding to each protein residue and should represent the overall structural homology between these proteins. Consistent with the methodology of Puente-Lelievre et al. we used trimAl^77^ with a gap threshold of 35% to create trimmed versions of the 3Di MSAs. In addition to the 3Di character alignments, we replaced the 3Di characters with the protein amino acid residues in both the complete and trimmed versions of the MSAs. This resulted in a total of four MSAs (3Di, trimmed 3Di, amino acid, trimmed amino acid) for each E, E1 and E2. Modelfinder^79^ implemented in IQ-TREE 2^78^ was used to determine the best substitution model for each alignment. All possible models were tested, including the custom 3Di substitution model (-mset Blosum62,Dayhoff,DCMut,JTT,JTTDCMut,LG,Poisson,Poisson+FQ,Poisson,PMB,WAG,EX2,EX3,EHO,EX_EHO,3DI -mfreq FU,F -mrate E,G,R). Selected substitution models for all alignments are included in the associated Zenodo repository (10.5281/zenodo.11092288 (ref. ^66^)). Phylogenetic trees based on each MSA were inferred using IQ-TREE 2 (v2.2.2.6)^78^ under each corresponding best-fit substitution model, with node support assessed using 1000 ultrafast bootstrap replicates^82^. Finally, we performed phylogenetic inferences based on both 3Di character and amino acid homology by combining the corresponding pairs of 3Di and amino acid MSAs and performing a partition model IQ-TREE 2 phylogenetic inference^104^, in which the two partitions correspond to the 3Di and the amino acid MSAs and each partition uses the best-fit substitution model of its corresponding MSA. The contribution of each partition in the combined MSA phylogenetic inferences was determined based on the partition-wise log-likelihoods, inferred with IQ-TREE’s -wpl option. Manual inspection and analysis of partition contribution was used to select trees for display in Fig. 4 (all resulting phylogenetic trees are provided in Extended Data Figs. 9–11). In brief, the 3Di partition had a consistently larger contribution to the joint phylogenetic inference compared to the amino acid partition, although using both alignments (instead of either alone) generally aids phylogenetic reconstruction^45^. However, in the case of the E glycoprotein phylogeny, the contribution of the amino acid partition was markedly lower than that of the 3Di partition (Supplementary Table 4). Moreover, we found clear evidence of long branch attraction in the amino acid only reconstructions of the E phylogeny (Extended Data Fig. 9), and reasoned that these artefacts may carry over into the combined 3Di–amino acid reconstruction. Therefore, the 3Di-based phylogeny was selected for the E protein whereas 3Di–amino acid trees were used for E1 and E2 (Fig. 4).

As a point of comparison, additional structural phylogenies were generated from our custom full length glycoprotein structures using FoldTree^105^. For each structure set (E, E1 and E2 protein sets) phylogenies were inferred using three metrics FoldTree, LDDT and TM-score with default parameters (see Supplementary Fig. 9). However, given the limitations associated with the use of neighbour joining methods on structural distances (outlined in Puente-Lelievre et al.^45^), we reasoned that the 3Di-guided approach, outlined above, is likely to yield more robust results.

### Reporting summary

Further information on research design is available in the Nature Portfolio Reporting Summary linked to this article.

## Online content

Any methods, additional references, Nature Portfolio reporting summaries, source data, extended data, supplementary information, acknowledgements, peer review information; details of author contributions and competing interests; and statements of data and code availability are available at 10.1038/s41586-024-07899-8.

## Supplementary information

Supplementary Information This file contains Supplementary Note 1, Supplementary Figs. 1–9 and references, which detail the MUSCLE alignment analysis, FoldTree structural analysis and the complete NS5b and T2 ribonuclease RNA virus phylogenies.

Reporting Summary

Supplementary Table 1 *Flaviviridae* sequence metadata including clade designations and GenBank nucleotide accession numbers.

Supplementary Table 2 Combination of sequence alignment, quality trimming methods, and amino acid substitution models used to infer the NS5b phylogenies.

Supplementary Table 3 *Flaviviridae* host association and vector status metadata related to Fig. 2c.

Supplementary Table 4 Contribution of 3Di vs amino acid in the glycoprotein phylogeny partition models.

## Data Availability

All underlying data, including sequences and structures, are available on *Zenodo* at 10.5281/zenodo.11092288 (ref. ^66^). The virus sequences assembled from SRA mining in this study are available in the Third-Party Annotation Section of the DDBJ/ENA/GenBank databases under the accession numbers TPA: BK067806–BK067816.

## Code availability

Original code and scripts related to the phylogenetic analyses are available at 10.5281/zenodo.11092288.
