# Supplementary material for: Mapping glycoprotein structure reveals Flaviviridae evolutionary history
Source: Nature. 2024 Sep 4;633(8030):695–703. doi: 10.1038/s41586-024-07899-8 (PMC11410658; doi:10.1038/s41586-024-07899-8)
Supplement: Supplementary file 1 — This file contains Supplementary Note 1, Supplementary Figs. 1–9 and references, which detail the MUSCLE alignment analysis, FoldTree structural analysis and the complete NS5b and T2 ribonuclease RNA virus phylogenies. [file 41586_2024_7899_MOESM1_ESM.pdf]

nature portfolio

https://doi.org/10.1038/s41586-024-07899-8

Supplementary information

Mapping glycoprotein structure reveals *Flaviviridae* evolutionary history

In the format provided by the authors and unedited

Mapping glycoprotein structure reveals *Flaviviridae* evolutionary history

Supplementary Information Inventory
This file contains the following:
Supplementary Note
Supplementary References
Supplementary Figures
Supplementary Tables (provided as individual .xlsx files)

SUPPLEMENTARY NOTE

Supplementary Note 1: MUSCLE alignment analysis

To demonstrate the robustness of the NS5b alignment and the resulting phylogenies we created a stratified MUSCLE alignment ensemble with the same NS5b sequences used to build the representative *Flaviviridae* phylogeny (Tree 18). The stratified ensemble comprised four replicates for different HMM parameters and four for guide tree merge order permutations, resulting in 16 total alignments (see Edgar, 2022). We measured the dispersion (D), the average distance between these replicate alignments, and found it to be 0.1320, thereby reflecting the presence of regions of low sequence conservation that are vulnerable to misalignment (D = 0 signifies that replicates are identical). To evaluate whether D > 0 affects the robustness of the MSA and tree topology, we first evaluated the consistency of the core *Flaviviridae* RdRp motif columns across the stratified MUSCLE replicate alignments and compared these to Alignment 18. Using the Palmscan software (v1.0)[102], we extracted RdRp palm motifs from 448 of the 461 NS5b sequences in the *Flaviviridae* amino acid sequence set. Sequences without detectable palm motifs were confided to the LGF and PLUN groups, and Motif C was manually detected in these. The extracted RdRp palm motifs were then aligned using MUSCLE and used to identify sequence logos, which were compared to those identified in the Kitrinoviricota[102,103] to obtain boundaries for each motif (**Supplementary Fig. 2**). We calculated letter confidence—a measure of how consistently a letter at a specific position is aligned—using the stratified MUSCLE replicates as input with the untrimmed Alignment 18 as the reference. Averaging across sequences, all positions within the palm motifs achieved the maximum possible letter confidence score, with the exception for position 2016 within motif B, which scored 8/9. Hence, high level of letter confidence suggests that despite the observed dispersion in replicates, the core motifs remain unaffected (**Supplementary Fig. 2**).

We next evaluated how dispersion (D) affects deep tree topology. Notably, the majority (56%, 9/16) of phylogenies estimated from the untrimmed MUSCLE replicates produced similar topologies to Tree 18, with the *Flaviviridae* placed into three distinct clades: (i) large genome flaviviruses and *Pestivirus*, (ii) *Orthoflavivirus*-*Jingmenvirus*, and (iii) *Pegivirus*-*Hepacivirus*. Two other topologies were also observed: (1) Scenario B in **Supplementary Fig. 6**, occurred in 38% (6/16) of phylogenies and is similar to that of Tree 18 with the exception that the pestiviruses fall basal to the *Orthoflavivirus*, *Jingmenvirus* and LGF: (2) Scenario C in **Supplementary Fig. 6** was only observed once. This phylogeny placed the pestiviruses basal to the hepaci- and pegiviruses. These alternative topologies/scenarios for *Flaviviridae* evolution were considered in further detail below. There was no pattern according to alignment length, pairwise identity, percentage identical sights and topology.

Phylogenetic analysis of divergent families such as the *Flaviviridae* typically employ quality trimming to remove poorly aligned regions from alignments before estimating phylogenies. To replicate this and the initial analysis used to select Tree 18, we trimmed each of the 16 MUSCLE replicates using trimAl with three gap thresholds (0.7, 0.8, and 0.9), eight consensus values (5, 7.5, 10, 12.5, 15, 17.5, 20, and 25), and one automated parameter selection mode (gappyout), resulting in 416 alignments (including the 16 untrimmed). Phylogenies were then estimated using three different substitution models resulting in a total of 1248 phylogenies. Neither a treespace PCoA analysis, nor mapping the distances between phylogenies in an MDS plot revealed clear clustering of the various guide tree merge order variations and HMM parameters, suggesting that the effects of dispersion from these perturbations is not easily predicted (**Supplementary Fig. 4**). We then repeated this in combination with the original phylogeny set constructed with MUSCLE and MAFFT finding that the original tree set (without ClustalO alignments) sits within the core diversity of the trees estimated from the stratified MUSCLE replicates (**Supplementary Fig. 5a & b**). This suggests our original analysis set covers much of the tree space explored by the MUSCLE stratified perturbations, and that both have a consistent core grouping.

Our estimation of the *Flaviviridae* phylogeny is particularly concerned with the deep evolutionary history of the family. We then sought to assess whether these phylogenies were consistent with the deep tree topology observed in our original analysis. After removing unclassified tips (i.e., those with clade labels PLUN, FJUN or HPUN [n = 19] in **Supplementary Table 1**) as these were typically on long branches and had highly inconsistent topologies, we assessed whether monophyly was present across various clade groupings consistent with our original phylogeny using the R package MonoPhy (v1.3)[104]. We found that 58% of the phylogenies matched Scenario A in the deep branching order (**Supplementary Fig. 6**). Notably, the LGF-Pestivirus clade was responsible for 99.8% (527/528) of deviations from Scenario A, with Scenario B the most commonly observed (38%) (**Supplementary Fig. 6**). Geometric median phylogenies created for the levels of HMM and guide tree perturbations showed similar topological ratios (Scenario A 56%, Scenario B 44%).

The varying frequencies of each topology across the guide tree and HMM parameters suggest that both contribute bias to the downstream analysis. For example, different topologies were consistently observed across the levels of HMM parameters when guide tree selection was held consistent (**Supplementary Fig. 3**). There is an exception to this; in phylogenies estimated from untrimmed MUSCLE replicates HMM level 3 and Guide tree variation level "none", all reflect the topology of Scenario A. This was also noted in the trimmed variations, where Scenario A was more consistently observed when these parameters were selected. Overall, the ratios of topologies across the parameter levels were similar to those observed in the untrimmed trees, suggesting that while trimming can reduce noise it appeared to have a limited effect on the deep branching order compared to the effects of parameters perturbed by MUSCLE.

Critically, therefore, despite the challenges of creating alignments and phylogenies over such extensive evolutionary spans, that our alignments and phylogenies almost exclusively reflect one of two deep topologies that only differ in the placement of the pestiviruses by a single node.

We argue that there are several key pieces of evidence that suggest Scenario A is the most parsimonious topology for the *Flaviviridae*:

1. Scenario A was the most common topology observed across aligners MAFFT and MUSCLE

In our original analysis we used multiple aligners to construct MSA. We note that Scenario A was the tree topology most commonly observed in both phylogenies estimated from MUSCLE (97%) and MAFFT (88%) alignments.

Of note, 60% of ClustalO phylogenies were consistent with Scenario B. In our initial analysis we found that several of the ClustalO topologies were discordant both in manual topological comparison and distance-based metrics: for example, the genus *Pegivirus* appeared in the middle of the hepaciviruses, while the *Hepacivirus* and *Pegivirus* clade would occasionally appear sister to the orthoflavi- and jingmenviruses. It is interesting to note that in benchmarks testing sequence aligners against simulated viral sequence data ClustalO performed poorly on sequences modelled on Hepatitis C virus compared to MUSCLE and MAFFT [105]. Due to these inconsistencies, we made the decision to exclude these alignments from our final tree selection in the original analysis.

2. Scenario A is consistent with the previously published *Flaviviridae* phylogenies despite methodological variations between studies. While studies presenting family level NS5b phylogenies that include representatives from the jingmenviruses and LGF are rare, where they have been constructed they all follow the same deep branching topology as Scenario A despite methodological differences [7,10,12,44,71,106]

3. **Scenario A is the most parsimonious biologically**. Scenario B assumes that the LGF, jingmenviruses and orthoflavivirues (including orthoflavi-like) diverged from a Pestivirus. Notably, orthoflavi-like viruses and endogenous viral elements (EVEs) have been identified in basal metazoans, including Cnidaria, suggesting a divergence from a flavi-like ancestor between 652-973 mya[44]. In contrast, which exclusively infect vertebrates, exhibit a clear pattern of virus-host co-divergence[47] and have no identified EVEs outside of mammals appear to have diverged more recently, 450-497 million years ago[44]. Given the ancient association of the orthoflavi-like viruses and that most vertebrate virus groups fall within larger invertebrate virus clades[107] it is unlikely that the LGF, orthoflaviviruses and jingmenviruses emerged from an ancestral vertebrate infecting Pestivirus. However, a scenario where this ancestral virus infected invertebrates cannot be entirely ruled out, despite the absence of evidence for a vast, unsampled viral diversity or EVEs along this branch. It is important to note that the Spider pesti-like viruses don’t fulfil this role. In Scenario B, where the pestiviruses fall basal to the LGF, the Spider pesti-like viruses typically cluster with the LGF rather than the pestiviruses. Lastly, the presence of a homologous RNase T2 in the pestiviruses and some members LGF but not in the jingmen- and orthoflaviviruses is provides further evidence for Scenario A. Taken together, it is more likely that the pestiviruses came from an invertebrate associated LGF-like ancestor (Scenario A) given the enormous diversity of these viruses and large gene pool available.

SUPPLEMENTARY REFERENCES

10. Paraskevopoulou, S. *et al.* Viromics of extant insect orders unveil the evolution of the flavi-like superfamily. *Virus Evolution* **7**, (2021).
13. Petrone, M. E. *et al.* A 39.8kb flavi-like virus uses a novel strategy for overcoming the RNA virus error threshold. *bioRxiv* 2024.01.08.574764 (2024) doi:10.1101/2024.01.08.574764.
15. Shi, M. *et al.* Divergent Viruses Discovered in Arthropods and Vertebrates Revise the Evolutionary History of the Flaviviridae and Related Viruses. *J. Virol.* **90**, 659–669 (2016).
47. Bamford, C. G. G., de Souza, W. M., Parry, R. & Gifford, R. J. Comparative analysis of genome-encoded viral sequences reveals the evolutionary history of flavivirids (family Flaviviridae). *Virus Evolution* **8**, (2022).
50. Mifsud, J. C. O. *et al.* Transcriptome mining extends the host range of the Flaviviridae to non-bilaterians. *Virus Evolution* **9**, (2022).
75. Dong, X. *et al.* A Novel Virus of Flaviviridae Associated with Sexual Precocity in Macrobrachium rosenbergii. *mSystems* **6**, e00003–21 (2021).
102. Babaian, A. & Edgar, R. Ribovirus classification by a polymerase barcode sequence. *PeerJ* **10**, e14055 (2022).
103. Charon, J., Buchmann, J. P., Sadiq, S. & Holmes, E. C. RdRp-scan: A bioinformatic resource to identify and annotate divergent RNA viruses in metagenomic sequence data. *Virus Evol* **8**, veac082 (2022).
104. Schwery, O. & O'Meara, B. C. MonoPhy: a simple R package to find and visualize monophyly issues. *PeerJ Computer Science* **2**, e56 (2016).
105. Young, C., Meng, S. & Moshiri, N. An Evaluation of Phylogenetic Workflows in Viral Molecular Epidemiology. *Viruses* **14**, (2022).
106. Dahan, J. *et al.* A Novel Flavi-like Virus in Alfalfa (Medicago sativa L.) Crops along the Snake River Valley. *Viruses* **14**, (2022).
107. Shi, M. *et al.* Redefining the invertebrate RNA virosphere. *Nature* **540**, 539–543 (2016).

SUPPLEMENTARY FIGURES

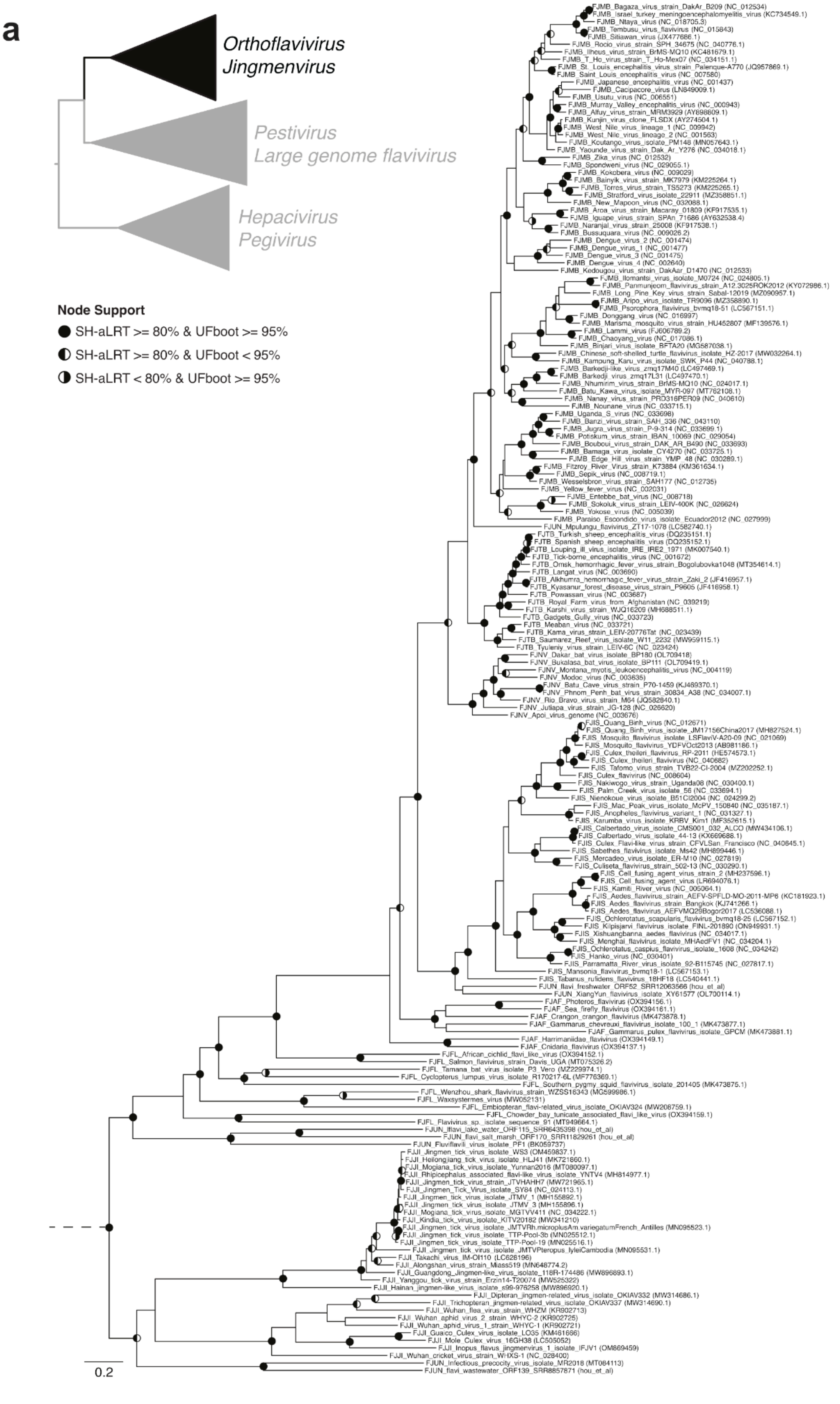

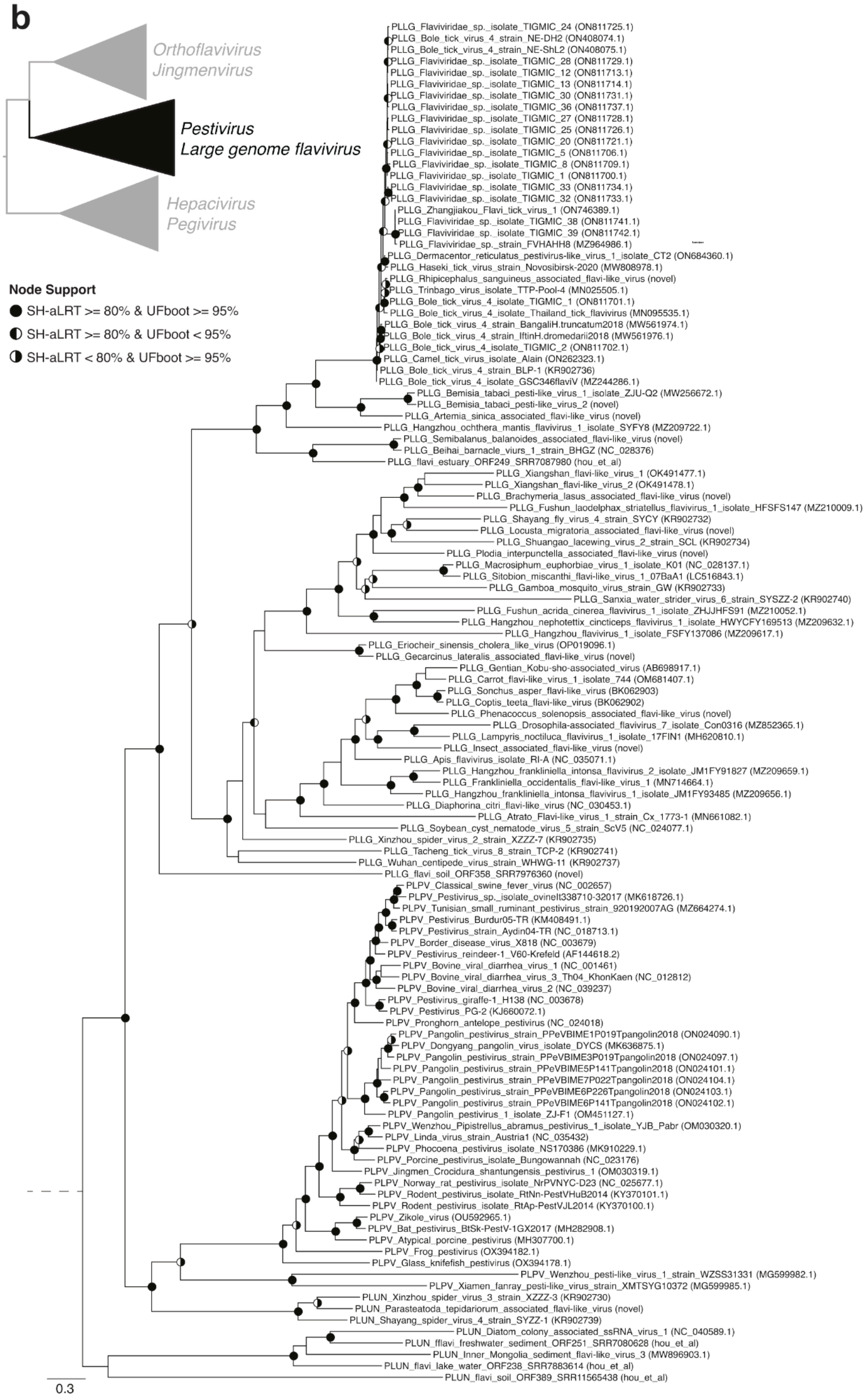

b
Orthoflavivirus
Jingmenvirus
Pestivirus
Large genome flavivirus
Hepacivirus
Pegivirus
Node Support
SH-aLRT >= 80% & UFboot >= 95%
SH-aLRT >= 80% & UFboot < 95%
SH-aLRT < 80% & UFboot >= 95%
PLLG_Flaviviridae_sp._isolate_TIGMIC_24 (ON811725.1)
PLLG_Bole_tick_virus_4_strain_NE-DH2 (ON408074.1)
PLLG_Bole_tick_virus_4_strain_NE-ShL2 (ON408075.1)
PLLG_Flaviviridae_sp._isolate_TIGMIC_28 (ON811729.1)
PLLG_Flaviviridae_sp._isolate_TIGMIC_12 (ON811713.1)
PLLG_Flaviviridae_sp._isolate_TIGMIC_13 (ON811714.1)
PLLG_Flaviviridae_sp._isolate_TIGMIC_30 (ON811731.1)
PLLG_Flaviviridae_sp._isolate_TIGMIC_36 (ON811737.1)
PLLG_Flaviviridae_sp._isolate_TIGMIC_27 (ON811728.1)
PLLG_Flaviviridae_sp._isolate_TIGMIC_25 (ON811726.1)
PLLG_Flaviviridae_sp._isolate_TIGMIC_20 (ON811721.1)
PLLG_Flaviviridae_sp._isolate_TIGMIC_5 (ON811706.1)
PLLG_Flaviviridae_sp._isolate_TIGMIC_8 (ON811709.1)
PLLG_Flaviviridae_sp._isolate_TIGMIC_1 (ON811700.1)
PLLG_Flaviviridae_sp._isolate_TIGMIC_33 (ON811734.1)
PLLG_Flaviviridae_sp._isolate_TIGMIC_32 (ON811733.1)
PLLG_Zhangjiakou_Flavi_tick_virus_1 (ON746389.1)
PLLG_Flaviviridae_sp._isolate_TIGMIC_38 (ON811741.1)
PLLG_Flaviviridae_sp._isolate_TIGMIC_39 (ON811742.1)
PLLG_Flaviviridae_sp._strain_FVHAHH8 (MZ964986.1)
PLLG_Dermacentor_reticulatus_pestivirus-like_virus_1_isolate_CT2 (ON684360.1)
PLLG_Haseki_tick_virus_strain_Novosibirsk-2020 (MW808978.1)
PLLG_Rhipicephalus_sanguineus_associated_flavi-like_virus (novel)
PLLG_Trinbago_virus_isolate_TTP-Pool-4 (MN025505.1)
PLLG_Bole_tick_virus_4_isolate_TIGMIC_1 (ON811701.1)
PLLG_Bole_tick_virus_4_isolate_Thailand_tick_flavivirus (MN095535.1)
PLLG_Bole_tick_virus_4_strain_BangaliH.truncatum2018 (MW561974.1)
PLLG_Bole_tick_virus_4_strain_IftinH.dromedarii2018 (MW561976.1)
PLLG_Bole_tick_virus_4_isolate_TIGMIC_2 (ON811702.1)
PLLG_Camel_tick_virus_isolate_Alain (ON262323.1)
PLLG_Bole_tick_virus_4_strain_BLP-1 (KR902736)
PLLG_Bole_tick_virus_4_isolate_GSC346flaviV (MZ244286.1)
PLLG_Bemisia_tabaci_pesti-like_virus_1_isolate_ZJU-Q2 (MW256672.1)
PLLG_Bemisia_tabaci_pesti-like_virus_2 (novel)
PLLG_Artemia_sinica_associated_flavi-like_virus (novel)
PLLG_Hangzhou_ochthera_mantis_flavivirus_1_isolate_SYFY8 (MZ209722.1)
PLLG_Semibalanus_balanoides_associated_flavi-like_virus (novel)
PLLG_Beihai_barnacle_viurs_1_strain_BHGZ (NC_028376)
PLLG_flavi_estuary_ORF249_SRR7087980 (hou_et_al)
PLLG_Xiangshan_flavi-like_virus_1 (OK491477.1)
PLLG_Xiangshan_flavi-like_virus_2 (OK491478.1)
PLLG_Brachymeria_lasus_associated_flavi-like_virus (novel)
PLLG_Fushun_laodelphax_striatellus_flavivirus_1_isolate_HFSFS147 (MZ210009.1)
PLLG_Shayang_fly_virus_4_strain_SYCY (KR902732)
PLLG_Locusta_migratoria_associated_flavi-like_virus (novel)
PLLG_Shuangao_lacewing_virus_2_strain_SCL (KR902734)
PLLG_Plodia_interpunctella_associated_flavi-like_virus (novel)
PLLG_Macrosiphum_euphorbiae_virus_1_isolate_K01 (NC_028137.1)
PLLG_Sitobion_miscanthi_flavi-like_virus_1_07BaA1 (LC516843.1)
PLLG_Gamboa_mosquito_virus_strain_GW (KR902733)
PLLG_Sanxia_water_strider_virus_6_strain_SYSZZ-2 (KR902740)
PLLG_Fushun_acrida_cinerea_flavivirus_1_isolate_ZHJJHFS91 (MZ210052.1)
PLLG_Hangzhou_nephotettix_cincticeps_flavivirus_1_isolate_HWYCFY169513 (MZ209632.1)
PLLG_Hangzhou_flavivirus_1_isolate_FSFY137086 (MZ209617.1)
PLLG_Eriocheir_sinensis_cholera_like_virus (OP019096.1)
PLLG_Gecarcinus_lateralis_associated_flavi-like_virus (novel)
PLLG_Gentian_Kobu-sho-associated_virus (AB698917.1)
PLLG_Carrot_flavi-like_virus_1_isolate_744 (OM681407.1)
PLLG_Sonchus_asper_flavi-like_virus (BK062903)
PLLG_Coptis_teeta_flavi-like_virus (BK062902)
PLLG_Phenacoccus_solenopsis_associated_flavi-like_virus (novel)
PLLG_Drosophila-associated_flavivirus_7_isolate_Con0316 (MZ852365.1)
PLLG_Lampyris_noctiluca_flavivirus_1_isolate_17FIN1 (MH620810.1)
PLLG_Insect_associated_flavi-like_virus (novel)
PLLG_Apis_flavivirus_isolate_RI-A (NC_035071.1)
PLLG_Hangzhou_frankliniella_intonsa_flavivirus_2_isolate_JM1FY91827 (MZ209659.1)
PLLG_Frankliniella_occidentalis_flavi-like_virus_1 (MN714664.1)
PLLG_Hangzhou_frankliniella_intonsa_flavivirus_1_isolate_JM1FY93485 (MZ209656.1)
PLLG_Diaphorina_citri_flavi-like_virus (NC_030453.1)
PLLG_Atrato_Flavi-like_virus_1_strain_Cx_1773-1 (MN661082.1)
PLLG_Soybean_cyst_nematode_virus_5_strain_ScV5 (NC_024077.1)
PLLG_Xinzhou_spider_virus_2_strain_XZZZ-7 (KR902735)
PLLG_Tacheng_tick_virus_8_strain_TCP-2 (KR902741)
PLLG_Wuhan_centipede_virus_strain_WHWG-11 (KR902737)
PLLG_flavi_soil_ORF358_SRR7976360 (novel)
PLPV_Classical_swine_fever_virus (NC_002657)
PLPV_Pestivirus_sp._isolate_ovineIt338710-32017 (MK618726.1)
PLPV_Tunisian_small_ruminant_pestivirus_strain_920192007AG (MZ664274.1)
PLPV_Pestivirus_Burdur05-TR (KM408491.1)
PLPV_Pestivirus_strain_Aydin04-TR (NC_018713.1)
PLPV_Border_disease_virus_X818 (NC_003679)
PLPV_Pestivirus_reindeer-1_V60-Krefeld (AF144618.2)
PLPV_Bovine_viral_diarrhea_virus_1 (NC_001461)
PLPV_Bovine_viral_diarrhea_virus_3_Th04_KhonKaen (NC_012812)
PLPV_Bovine_viral_diarrhea_virus_2 (NC_039237)
PLPV_Pestivirus_giraffe-1_H138 (NC_003678)
PLPV_Pestivirus_PG-2 (KJ660072.1)
PLPV_Pronghorn_antelope_pestivirus (NC_024018)
PLPV_Pangolin_pestivirus_strain_PPeVBIME1P019Tpangolin2018 (ON024090.1)
PLPV_Dongyang_pangolin_virus_isolate_DYCS (MK636875.1)
PLPV_Pangolin_pestivirus_strain_PPeVBIME3P019Tpangolin2018 (ON024097.1)
PLPV_Pangolin_pestivirus_strain_PPeVBIME5P141Tpangolin2018 (ON024101.1)
PLPV_Pangolin_pestivirus_strain_PPeVBIME7P022Tpangolin2018 (ON024104.1)
PLPV_Pangolin_pestivirus_strain_PPeVBIME6P226Tpangolin2018 (ON024103.1)
PLPV_Pangolin_pestivirus_strain_PPeVBIME6P141Tpangolin2018 (ON024102.1)
PLPV_Pangolin_pestivirus_1_isolate_ZJ-F1 (OM451127.1)
PLPV_Wenzhou_Pipistrellus_abramus_pestivirus_1_isolate_YJB_Pabr (OM030320.1)
PLPV_Linda_virus_strain_Austria1 (NC_035432)
PLPV_Phocoena_pestivirus_isolate_NS170386 (MK910229.1)
PLPV_Porcine_pestivirus_isolate_Bungowannah (NC_023176)
PLPV_Jingmen_Crocidura_shantungensis_pestivirus_1 (OM030319.1)
PLPV_Norway_rat_pestivirus_isolate_NrPVNYC-D23 (NC_025677.1)
PLPV_Rodent_pestivirus_isolate_RtNn-PestVHuB2014 (KY370101.1)
PLPV_Rodent_pestivirus_isolate_RtAp-PestVJL2014 (KY370100.1)
PLPV_Zikole_virus (OU592965.1)
PLPV_Bat_pestivirus_BtSk-PestV-1GX2017 (MH282908.1)
PLPV_Atypical_porcine_pestivirus (MH307700.1)
PLPV_Frog_pestivirus (OX394182.1)
PLPV_Glass_knifefish_pestivirus (OX394178.1)
PLPV_Wenzhou_pesti-like_virus_1_strain_WZSS31331 (MG599982.1)
PLPV_Xiamen_fanray_pesti-like_virus_strain_XMTSYG10372 (MG599985.1)
PLUN_Xinzhou_spider_virus_3_strain_XZZZ-3 (KR902730)
PLUN_Parasteatoda_tepidariorum_associated_flavi-like_virus (novel)
PLUN_Shayang_spider_virus_4_strain_SYZZ-1 (KR902739)
PLUN_Diatom_colony_associated_ssRNA_virus_1 (NC_040589.1)
PLUN_fflavi_freshwater_sediment_ORF251_SRR7080628 (hou_et_al)
PLUN_Inner_Mongolia_sediment_flavi-like_virus_3 (MW896903.1)
PLUN_flavi_lake_water_ORF238_SRR7883614 (hou_et_al)
PLUN_flavi_soil_ORF389_SRR11565438 (hou_et_al)
0.3

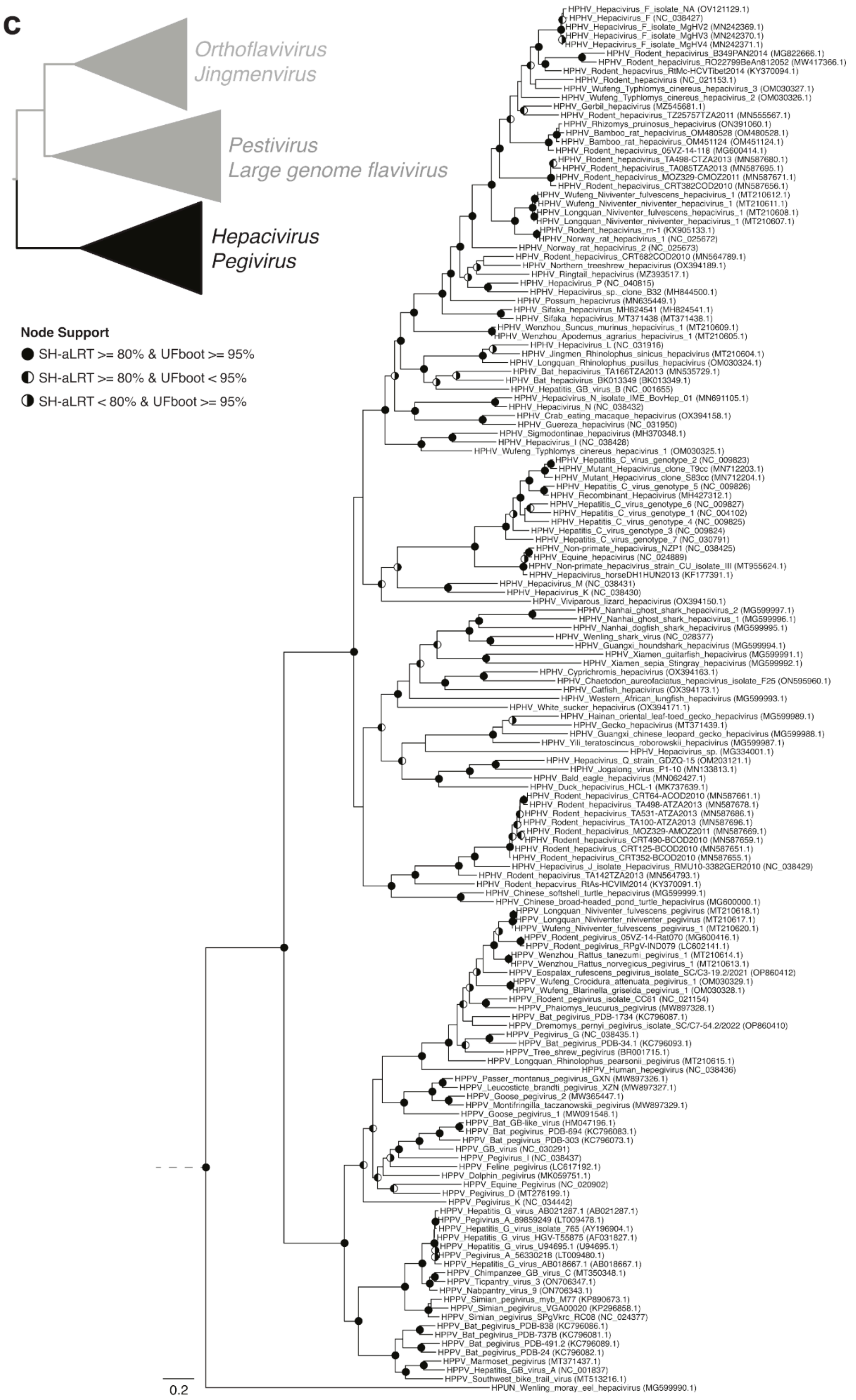

Supplementary Figure 1. NS5b consensus phylogeny (Tree 18) with tip labels. The complete NS5b phylogeny (underlying Fig. 1a and 2a) split by the major *Flaviviridae* lineages **a,** *Orthoflavivirus/Jingmenvirus*, **b,** Large genome flavivirus/*Pestivirus* and **c,** *Hepacivirus/Pegivirus*. A scale bar denotes the number of amino acid substitutions per site. Node support (SH-aLRT >=80% and UFboot >=95%) is indicated by a black circle while intermediate support (SH-aLRT <80% and UFboot >=95% or SH-aLRT >80% and UFboot <95%) is indicated by a half filled circle corresponding to the indice above the support threshold.

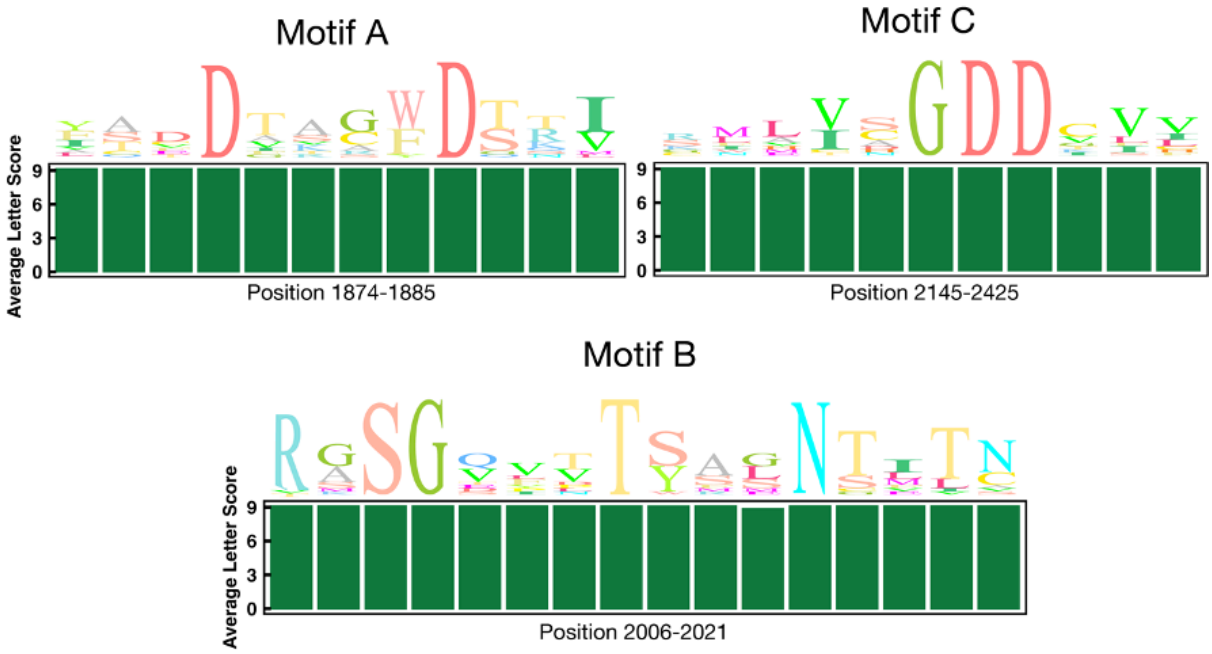

Supplementary Figure 2. Letter confidence measurements for the NS5b palmprint motifs (n = 448 sequences). An average of letter confidence, which ranges from 0 (low confidence) to 9 (high confidence), was calculated by comparing the position of each amino acid in the MUSCLE replicates to a reference alignment, untrimmed Tree 18 (flaviviridae_ns5_untrimmed_MUSCLE_20230630). The position of each motif in the untrimmed Tree 18 MUSCLE alignment is depicted under the barplot.

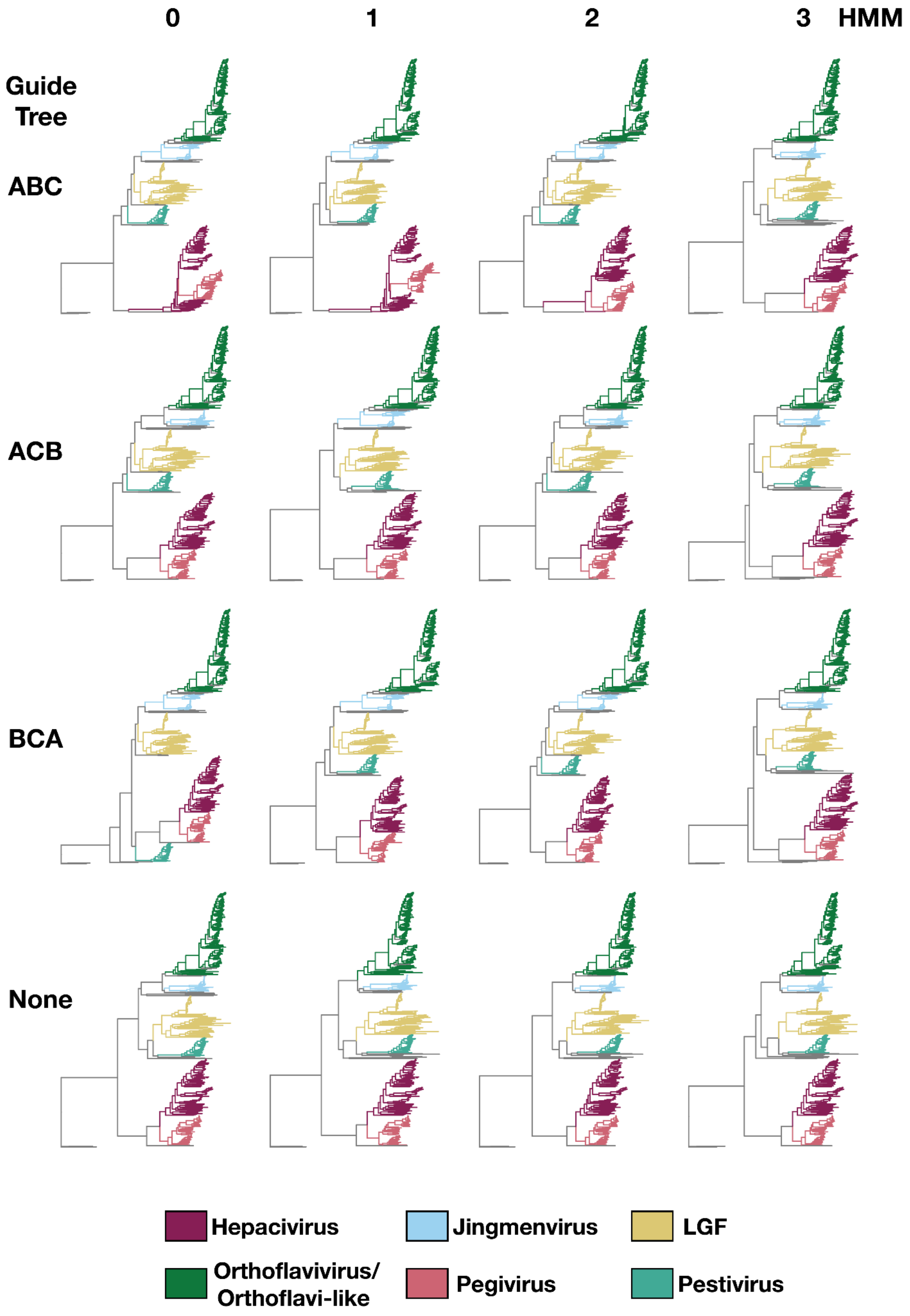

Supplementary Figure 3. NS5b phylogenies estimated from the stratified MUSCLE replicates. Guide tree merge order is held fixed on one axis, and HMM parameters are held fixed on the other axis. Branches are coloured by *Flaviviridae* clade as defined in Supplementary Table 1. Tips corresponding to unclassified sequences (e.g, FJUN, PLUN and HPUN) were coloured black.

Phylogenies were inferred using IQTREE 2 and the LG+F+R10 amino acid substitution model. Phylogenies are rooted on the *Tombusvirus* outgroup.

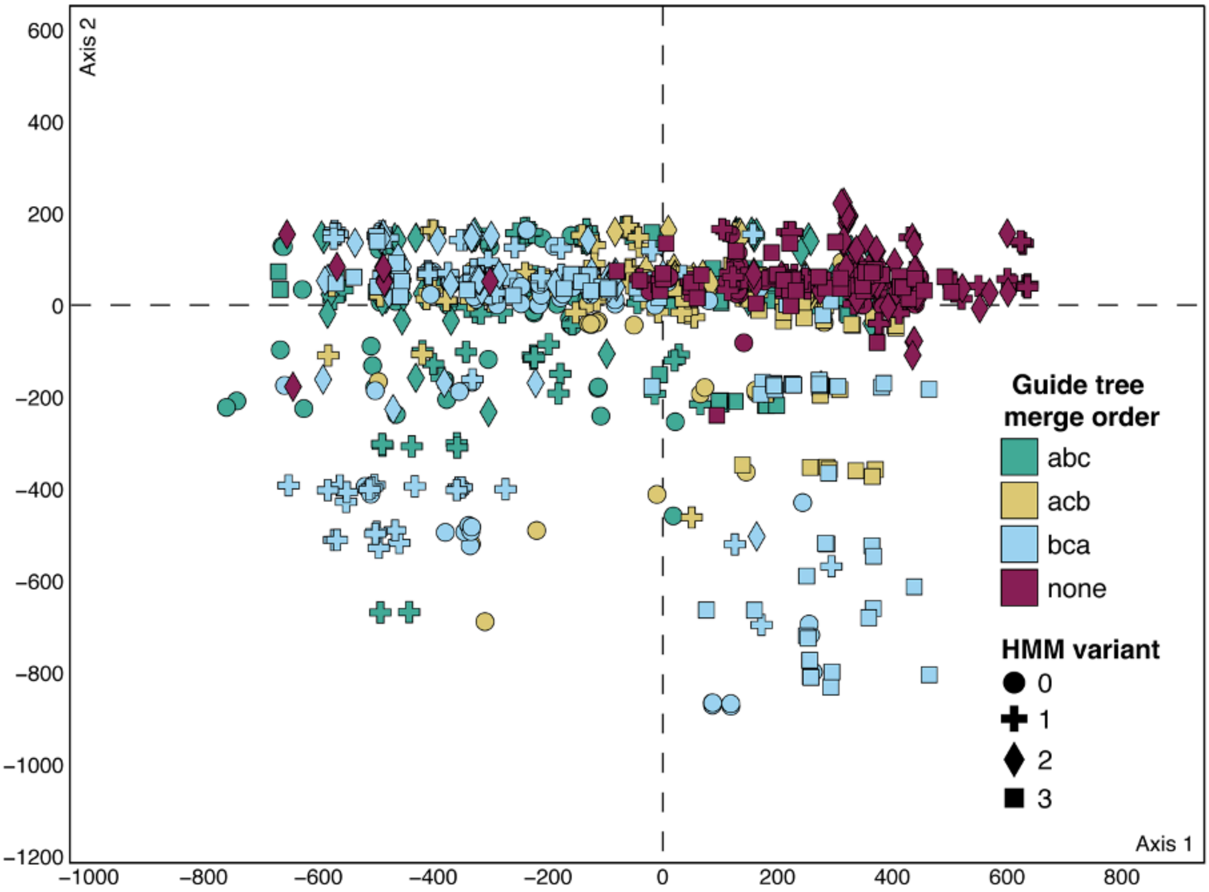

Supplementary Figure 4. Two-dimensional MDS plot of the NS5b stratified MUSCLE replicate phylogenies. Points are coloured by guide tree merge order and shapes indicate HMM parameters. Phylogeny none.1_cons17.5_gt0.7_20240420_LG.treefile was excluded from the scatterplot as it was a clear outlier.

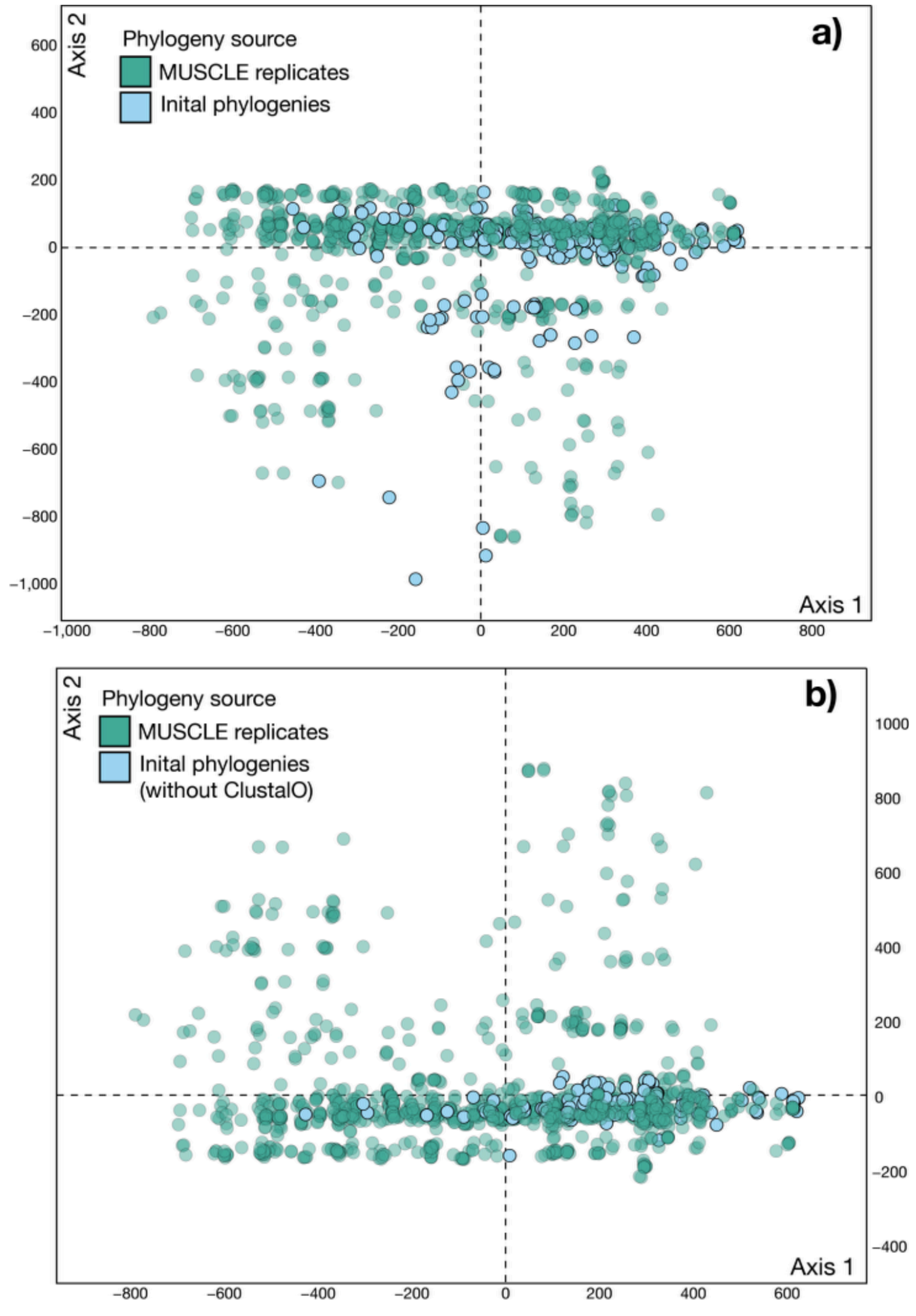

Supplementary Figure 5. Two-dimensional MDS plot of the NS5b stratified MUSCLE replicate phylogenies. **a,** Coloured by whether they were generated in the initial alignment analysis or from the stratified MUSCLE replicates **b,** Excluding trees generated from Clustal Omega alignments.none.1_cons17.5_gt0.7_20240420_LG.treefile was excluded from the scatterplot as it was a clear outlier.

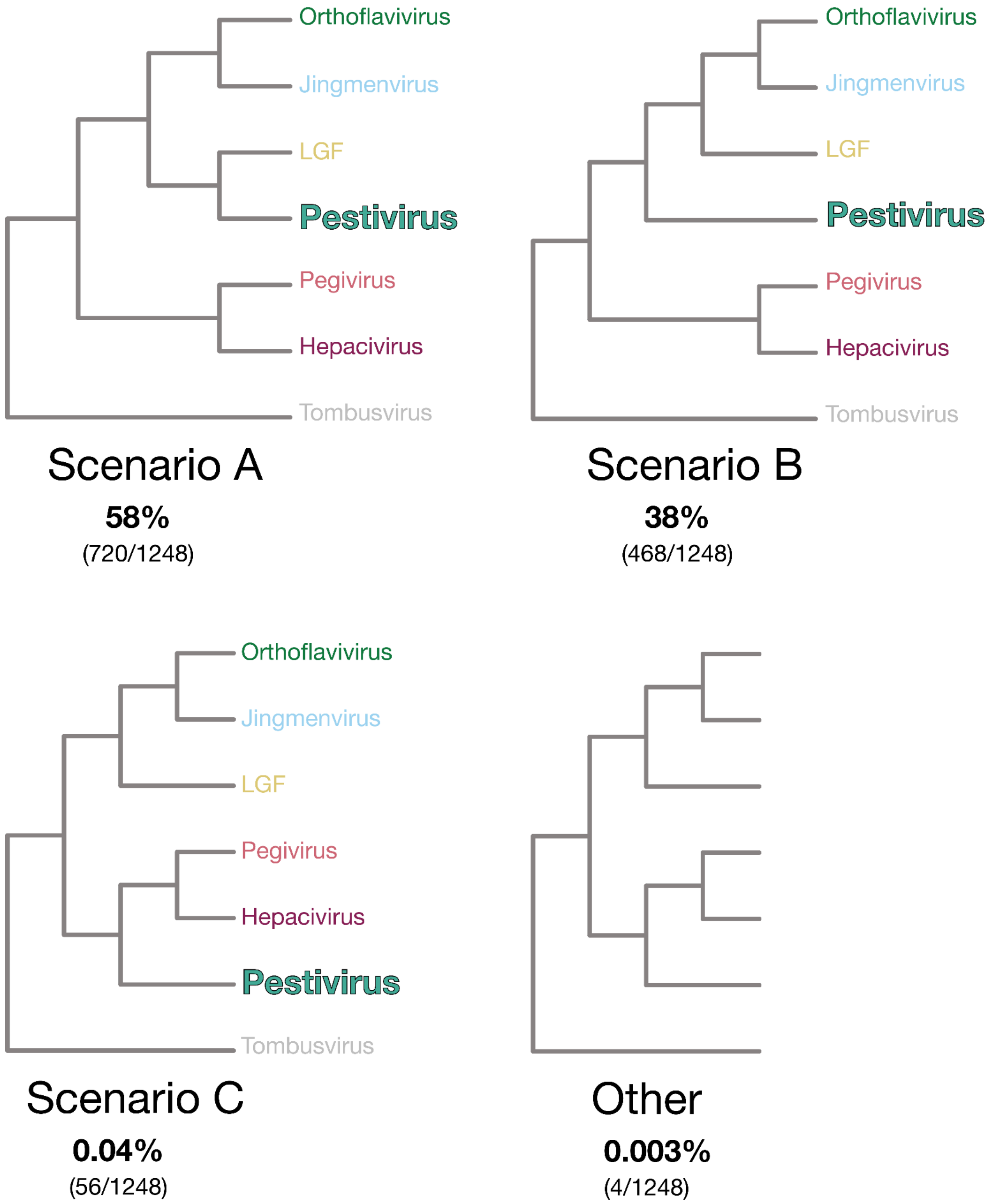

Supplementary Figure 6. Illustrative cladogram showing the major *Flaviviridae* clades topologies observed in trees estimated from the stratified MUSCLE alignment replicates. Each clade is individually coloured and the Pestiviruses bolded to highlight the differences in topology between the scenarios. Under each scenario the percentage and number of phylogenies it was observed in are shown.

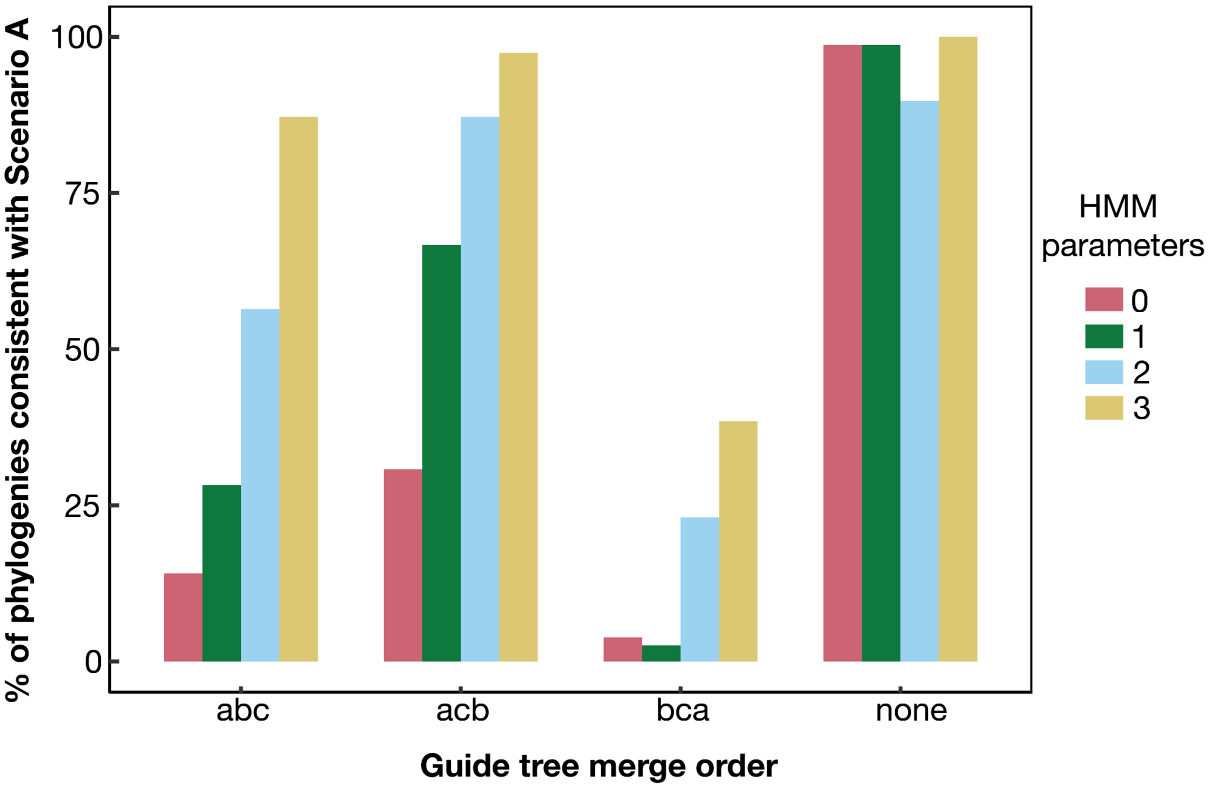

Supplementary Figure 7. Barplot showing the percentage of NS5b stratified MUSCLE replicate phylogenies that are consistent with the deep branching topology of Tree 18 (Scenario A, Supplementary Fig 5) across guide tree merge order levels and coloured by HMM parameter choice.

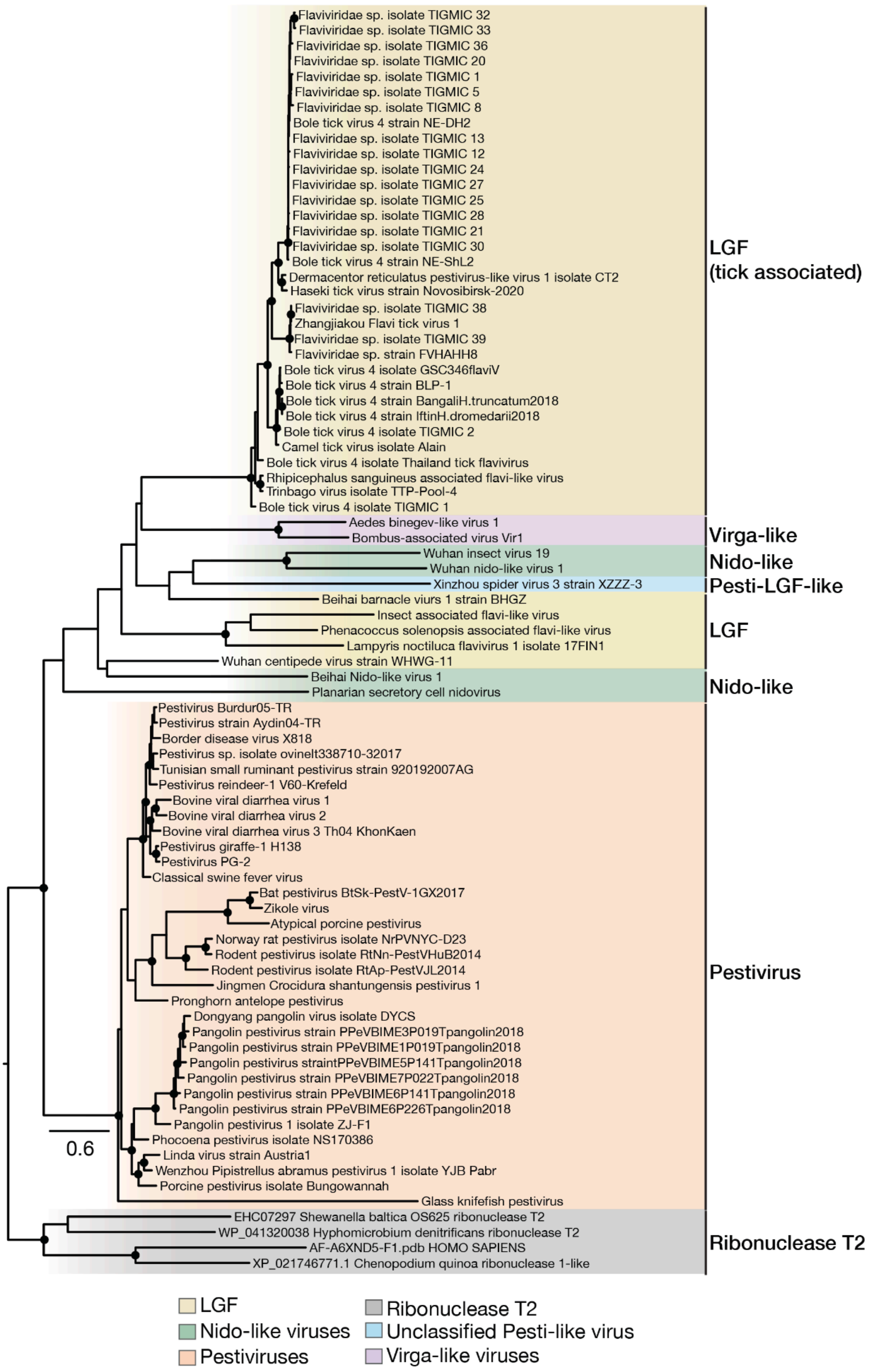

Supplementary Figure 8. Phylogeny of the RNA virus RNase T2/Erns clade rooted on non-viral sequences, with viral clades colour-coded as shown in the key. A scale bar denotes the number of amino acid substitutions per site. Node support (SH-aLRT >=80% and UFboot >=95%) is indicated by a black circle.

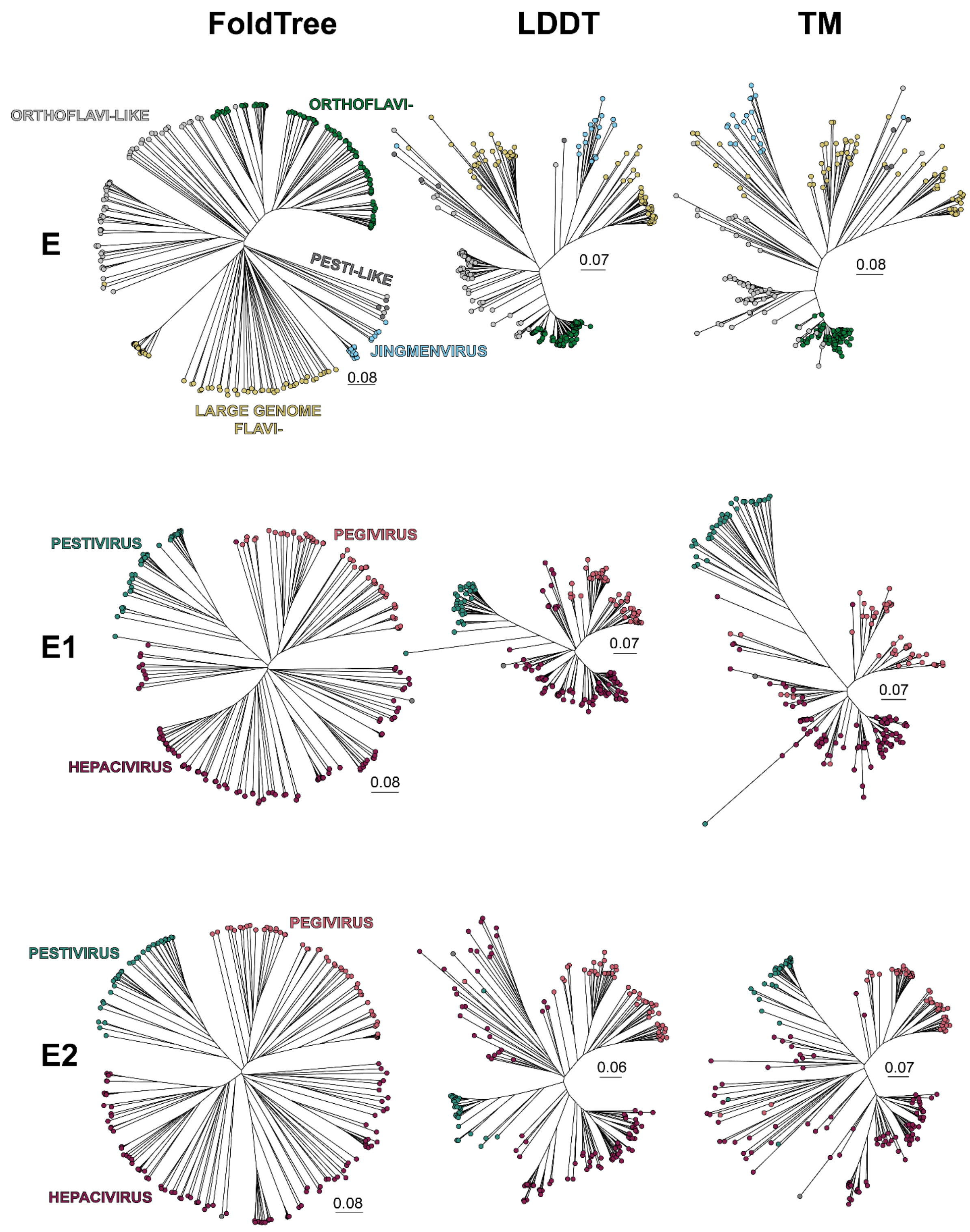

Supplementary Figure 9. Structurally aligned glycoprotein phylogenies using FoldTree. For each glycoprotein (E, E1 and E2) phylogenies generated using three different measures of structural distance, Foldtree metric, LDDT and TM score are shown. For visualisation the unrooted phylogenies with statistical correction are shown. Tip circles are coloured by *Flaviviridae* clade. PP unrooted trees chosen for visualisation. All resulting phylogenetic trees are provided in the associated Zenodo repository.

SUPPLEMENTARY TABLE (provided as individual .xlsx files)

Supplementary Table 1. *Flaviviridae* sequence metadata including clade designations and GenBank nucleotide accession numbers.
Supplementary Table 2. Combination of sequence alignment, quality trimming methods, and amino acid substitution models used to infer the NS5b phylogenies
Supplementary Table 3. *Flaviviridae* host association and vector status metadata related to Fig. 2c
Supplementary Table 4. Contribution of 3Di vs amino acid in the glycoprotein phylogeny partition models
