# Supplementary material for: Mapping glycoprotein structure reveals Flaviviridae evolutionary history
Source: Nature. 2024 Sep 4;633(8030):695–703. doi: 10.1038/s41586-024-07899-8 (PMC11410658; doi:10.1038/s41586-024-07899-8)
Supplement: Supplementary file 2 — Reporting Summary [file 41586_2024_7899_MOESM2_ESM.pdf]

nature portfolio

Corresponding author(s): Joe Grove
Last updated by author(s): Jul 7, 2024

Reporting Summary

Nature Portfolio wishes to improve the reproducibility of the work that we publish. This form provides structure for consistency and transparency in reporting. For further information on Nature Portfolio policies, see our Editorial Policies and the Editorial Policy Checklist.

Statistics

For all statistical analyses, confirm that the following items are present in the figure legend, table legend, main text, or Methods section.

n/a	Confirmed	
☒	☐	The exact sample size (*n*) for each experimental group/condition, given as a discrete number and unit of measurement
☒	☐	A statement on whether measurements were taken from distinct samples or whether the same sample was measured repeatedly
☒	☐	The statistical test(s) used AND whether they are one- or two-sided<br>*Only common tests should be described solely by name; describe more complex techniques in the Methods section.*
☒	☐	A description of all covariates tested
☒	☐	A description of any assumptions or corrections, such as tests of normality and adjustment for multiple comparisons
☐	☒	A full description of the statistical parameters including central tendency (e.g. means) or other basic estimates (e.g. regression coefficient) AND variation (e.g. standard deviation) or associated estimates of uncertainty (e.g. confidence intervals)
☒	☐	For null hypothesis testing, the test statistic (e.g. *F*, *t*, *r*) with confidence intervals, effect sizes, degrees of freedom and *P* value noted<br>*Give P values as exact values whenever suitable.*
☒	☐	For Bayesian analysis, information on the choice of priors and Markov chain Monte Carlo settings
☒	☐	For hierarchical and complex designs, identification of the appropriate level for tests and full reporting of outcomes
☒	☐	Estimates of effect sizes (e.g. Cohen's *d*, Pearson's *r*), indicating how they were calculated

Our web collection on statistics for biologists contains articles on many of the points above.

Software and code

Policy information about availability of computer code

Data collection: No software was used for data collection.

Data analysis:
AlphaFold (v2.3)
BatchArtemisSRAMiner (v1.0.3)
CD-HIT (v4.6.1)
Clustal Omega (v1.2.4)
ColabFold (v1.5.1)
DIAMOND (v2.0.9)
DIAMOND cluster (v2.0.9)
ESMFold (v1)
FATCAT (v2.0)
FigTree (v1.4.4)
FoldSeek (v53465f07cdeed1f7fda08ee7f188327cb57c37ba)
Geneious Prime (v2022.0)
HMM search webserver (v2.41.2)
InterProScan (v5.56-89.0, v5.63-95.0)
IQ-TREE (v2.1.0, v2.2.2.6)
Kingfisher (v0.3.0)
MAFFT (v7.402)
MEGAHIT (v.1.2.9)
MUSCLE (v5.1)

Python Package 'ETE3 (v3.1.3)' '
R Package 'ape (v.5.6-2)'
R Package 'gggenomes (v0.9.8.9)'
R Package 'ggtree (v3.3.0.9)'
R Package 'phytools (v1.5-1)'
R package 'Treespace (v1.1.4.2)'
Serratus (v0.3.5)
SignalP (v6.0)
TrimAl (v1.4.1)
Trimmomatic (v0.38)
Custom code can be found at https://zenodo.org/doi/10.5281/zenodo.10616317

For manuscripts utilizing custom algorithms or software that are central to the research but not yet described in published literature, software must be made available to editors and reviewers. We strongly encourage code deposition in a community repository (e.g. GitHub). See the Nature Portfolio guidelines for submitting code & software for further information.

Data

Policy information about availability of data

All manuscripts must include a data availability statement. This statement should provide the following information, where applicable:

- Accession codes, unique identifiers, or web links for publicly available datasets
- A description of any restrictions on data availability
- For clinical datasets or third party data, please ensure that the statement adheres to our policy

All underlying data, including sequences, structures and associated code, are available here: https://zenodo.org/doi/10.5281/zenodo.10616317
. The virus sequences assembled from SRA mining in this study are available in the Third Party Annotation Section of the DDBJ/ENA/GenBank databases under the accession numbers TPA: BK067806-BK067816. Flaviviridae nucleotide sequences were obtained through the NCBI Virus Database (https://www.ncbi.nlm.nih.gov/labs/virus/vssi/#/) and NCBI Nucleotide database (https://www.ncbi.nlm.nih.gov/nucleotide/). Additional novel virus genomes Hou et al (https://www.biorxiv.org/content/10.1101/2023.04.18.537342v2) (http://47.93.21.181/Results/RdRP_dataset/contig/Supergroup005_contig.fa). Libraries used to identitfy novel LGF-like viruses were obtained from the Sequence Read Archive (https://www.ncbi.nlm.nih.gov/sra). Host information and vector status was determined using the GenBank database(https://www.ncbi.nlm.nih.gov/genbank/) and Arbovirus Catalog (https://wwwn.cdc.gov/arbocat/).
Genome annotations through InterProScan used the SFLD (v4.0) (http://sfld.rbvi.ucsf.edu/archive/django/index.html), PANTHER (v17.0) (https://www.pantherdb.org), SuperFamily (https://supfam.org), PROSITE (https://prosite.expasy.org), CDD (https://www.ncbi.nlm.nih.gov/cdd/), Pfam (https://www.ebi.ac.uk/interpro/entry/pfam/#table), PRINTS (https://www.ebi.ac.uk/interpro/entry/prints/#table), CATH-Gene3D (https://www.ebi.ac.uk/interpro/entry/cathgene3d/#table), AntiFam (https://www.ebi.ac.uk/interpro/entry/antifam/#table), FunFAM (http://cathdb.info), MobiDBLite (https://mobidb.bio.unipd.it), NCBIfam (https://www.ebi.ac.uk/interpro/entry/ncbifam/#table), PIRSF (https://proteininformationresource.org/pirsf/), PDB (https://www.rcsb.org/#Category-welcome). To identify RNase T2-like sequences in virus genomes the NR clustered (https://ncbiinsights.ncbi.nlm.nih.gov/2022/05/02/clusterednr_1/), Reference Proteomes (https://www.ebi.ac.uk/reference_proteomes/), UniProtKB (https://www.uniprot.org/help/uniprotkb), SCOPe (https://scop.berkeley.edu), SMART (http://smart.embl-heidelberg.de), Pfam (https://www.ebi.ac.uk/interpro/entry/pfam/#table), PHROG (https://phrogs.lmge.uca.fr), COG (https://www.ncbi.nlm.nih.gov/research/cog), Uniclust30 (https://wwwuser.gwdguser.de/~compbiol/uniclust/2023_02/), AlphaFold Clusters (https://cluster.foldseek.com) and KOG (https://mycocosm.jgi.doe.gov/help/kogbrowser.jsf) databases were used. For flavivirus discovery the NCBI non-redundant protein database (https://ftp.ncbi.nlm.nih.gov/blast/db/FASTA/nr.gz) was used. Reference structures were obtained from 6ZQI (Spondweni virus E and prM) (https://www.rcsb.org/structure/6ZQI), 1L9K (DENV-2 MTase) (https://www.rcsb.org/structure/1L9K), 5F3Z (DENV-3 RdRp) (https://www.rcsb.org/structure/5F3Z), 7QRF (TBEV E and prM) (https://www.rcsb.org/structure/7QRF), 7V1E (Omsk hemorrhagic fever virus MTase) (https://www.rcsb.org/structure/7V1E), 7T6X (HCV E1 and E2) (https://www.rcsb.org/structure/7T6X), 6VYB (SARS-CoV-2 spike, negative control) (https://www.rcsb.org/structure/6VYB), 2YQ2 (BVDV E2) (https://www.rcsb.org/structure/2YQ2), 4DVK (BVDV Erns) (https://www.rcsb.org/structure/4DVK) and Oliver et al. (https://journals.plos.org/plosbiology/article?id=10.1371/journal.pbio.3002174) (https://zenodo.org/records/7221315).

Research involving human participants, their data, or biological material

Policy information about studies with human participants or human data. See also policy information about sex, gender (identity/presentation), and sexual orientation and race, ethnicity and racism.

Reporting on sex and gender	N/A
Reporting on race, ethnicity, or other socially relevant groupings	N/A
Population characteristics	N/A
Recruitment	N/A
Ethics oversight	N/A

Note that full information on the approval of the study protocol must also be provided in the manuscript.

Field-specific reporting

Please select the one below that is the best fit for your research. If you are not sure, read the appropriate sections before making your selection.

☐ Life sciences ☐ Behavioural & social sciences ☒ Ecological, evolutionary & environmental sciences

For a reference copy of the document with all sections, see nature.com/documents/nr-reporting-summary-flat.pdf

Ecological, evolutionary & environmental sciences study design

All studies must disclose on these points even when the disclosure is negative.

Study description	Phylogenetic and protein structure prediction analysis of Flaviviridae sequences to survey the distribution of glycoproteins. The study design is consistent with a discovery-oriented project.
Research sample	The taxonomic sample includes all Flaviviridae nucleotide sequences deemed to have complete coding sequences as of the 15th of December 2022. As additional Flaviviridae sequences became available during the course of the analysis those deemed relevant (e.g., those that fell in the boundaries of taxonomic groups or related to the Bole tick virus clade) to the current study were added. A complete list of these studies can be found in the Methods section. A complete list of all sequences can be found in Supplementary Table 1. In addition, the research sample includes virus sequences that were assembled from the reanalysis of Sequence Read Archive (SRA) data. SRA libraries were chosen based on results from the Serratus micro-assemblies (see Methods)
Sampling strategy	No sample-size calculations were performed. For our collection of Flaviviridae sequences we opted to search all available/relevant data available in the NCBI nucleotide and virus databases. For our search of SRA libraries we limited the number of libraries we reassembled based on a DIAMOND BLASTx e-value threshold of 1.6E−1. This was implemented to restrict the number of libraries for reassembly to a manageable quantity. This threshold was determined based on the organism associated with the SRA library and the percent identity values of the micro-assemblies to the Haseki tick virus query.
Data collection	Flavivirus sequences were compiled by Jonathon Mifsud using the search phrase "Flaviviridae taxid 11050 and Unclassified Flaviviridae taxid 38144" in the NCBI Virus Database and using the search phrase "flavi[All Fields] OR pesti[All Fields] OR hepaci[All Fields] OR pegi[All Fields] AND viruses[filter]" from the NCBI nucleotide database on the 15th of December 2022. In addition, Flavivirus sequences were obtained from various studies, see Methods for information on these studies. See Supplementary Table 1. for a complete list of all nucleotide sequences collected. In addition, several potentially novel flavivirus sequences formed part of the data collection. These were assembled from publicly available sequencing data uploaded to the SRA. For details regarding this, see the Methods and the Serratus_sra_summary_table table in the Zenodo data file https://zenodo.org/doi/10.5281/zenodo.10616317
Timing and spatial scale	Sequences from the NCBI nucleotide and virus databases were obtained on the 15th of December 2022. Sequences from the literature obtained after this date were collected as made available. Serratus microassemblies, which were used for the SRA search were downloaded in April 2023. Raw sequencing reads from the SRA libraries were downloaded in April-May 2023.
Data exclusions	Genome sequence data was excluded if it was deemed to be partial in genome completeness. This was chosen as including partial genomes would make it difficult to determine whether proteins were present in lineages or whether that part of the genome was not present. Furthermore, the vast majority of partial sequences are of the NS5 region and would likely contribute little to our analysis of glycoproteins.
Reproducibility	Data analyses are reproducible with the information given in the methods and the data present in Zenodo (https://zenodo.org/doi/10.5281/zenodo.10616317)
Randomization	No randomization was performed in this study and no controlling for covariants is not relevant to this studies design.
Blinding	Blinding does not apply to this study as it is discovery-oriented.

Did the study involve field work? ☐ Yes ☒ No

Reporting for specific materials, systems and methods

We require information from authors about some types of materials, experimental systems and methods used in many studies. Here, indicate whether each material, system or method listed is relevant to your study. If you are not sure if a list item applies to your research, read the appropriate section before selecting a response.

Materials & experimental systems

n/a	Involved in the study
☒	☐ Antibodies
☒	☐ Eukaryotic cell lines
☒	☐ Palaeontology and archaeology
☒	☐ Animals and other organisms
☒	☐ Clinical data
☒	☐ Dual use research of concern
☒	☐ Plants

Methods

n/a	Involved in the study
☒	☐ ChIP-seq
☒	☐ Flow cytometry
☒	☐ MRI-based neuroimaging

Plants

Seed stocks	N/A
Novel plant genotypes	N/A
Authentication	N/A
